# Study on the Reaction Mechanism of RuO_x_ and Its Enhanced Hydrogen Evolution Reaction Activity

**DOI:** 10.1002/advs.76132

**Published:** 2026-06-22

**Authors:** Liquan Zhang, Haobo Dong, Jianrui Feng, Longxiang Liu, Fei Guo, Liqun Kang, Guoliang Chai, Mohamed A. Ghanem, Xinde Chen, Ivan P. Parkin

**Affiliations:** ^1^ Department of Chemistry, Faculty of Mathematical and Physical Sciences University College London London UK; ^2^ Guangzhou Institute of Energy Conversion Chinese Academy of Sciences Guangzhou P. R. China; ^3^ School of Future Technology South China University of Technology Guangzhou P. R. China; ^4^ Department of Materials University of Oxford Oxford UK; ^5^ Max‐Planck‐Institut für Chemische Energiekonversion Mülheim an der Ruhr Deutschland; ^6^ State Key Laboratory of Structural Chemistry, Fujian Institute of Research on the Structure of Matter Chinese Academy of Sciences (CAS) Fujian China; ^7^ Department of Chemistry, College of Sciences King Saud University Riyadh Saudi Arabia

**Keywords:** hydrogen evolution reaction, reaction mechanism, ruthenium oxide, surface reconstruction, in situ raman

## Abstract

Ruthenium oxide is a well‐known electrocatalyst for water splitting. Despite many advantages, the reaction mechanism and the structure changes of ruthenium oxide are unclear during catalysis, and the catalytic efficiency of ruthenium oxides remains a concern. In this paper, a needle‐shaped copper substrate was synthesized, and the performance of RuO_x_ was optimized. Reversible in situ Raman signals were found during the reaction process, which confirmed from the reversible surface reconstruction of RuO_x_. Density Functional Theory calculations found that the actual catalytic species is Ru(II)O. A detailed reaction mechanism of the Volmer step on the surface of ruthenium oxide was proposed based on the findings. The results indicate that the needles can lower the overpotentials of RuO_x_. At a current density of 10 mA·cm 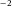, the overpotential of RuO_x_@ndl. was 68% lower than that of RuO_x_@b. f., which is much lower than the overpotential of commercial Pt(20wt.%)/C. This research provides a fundamental understanding of ruthenium oxide's reaction mechanism and gives insight into the design of the hydrogen evolution reaction catalysts.

## Graphical abstract

In situ Raman spectroscopy and DFT calculations reveal that, prior to the onset of HER, the surface of RuOx undergoes reversible reduction and structural reconstruction. The resulting Ru(II)O/Ru(IV)O2 configuration is identified as the true catalytically active species. Based on this reconstructed surface, a plausible Volmer reaction pathway is proposed in the schematic.

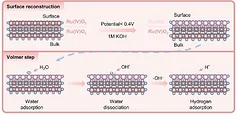

## 1 Introduction

One of the primary challenges in hydrogen production through electrochemical water splitting is enhancing energy conversion efficiency by reducing the reaction overpotential [1, 2, 3, 4]. Numerous catalysts for alkaline hydrogen evolution have been reported [5, 6, 7, 8, 9, 10]. Among these, ruthenium‐based catalysts have been extensively studied for their excellent performance in alkaline environments [11, 12, 13, 14, 15]. For example, Cao and co‐workers developed a WC_x_‐supported Ru nanoparticle catalyst, they found that an electron‐deficient Ru state could enhance alkaline hydrogen evolution reaction (HER) performance in Anion Exchange Membrane (AEM) electrolyzer [16]. Song and co‐workers demonstrated that Fe alloying in supported Ru catalysts enhances water dissociation and hydrogen adsorption for highly efficient alkaline HER [17]. Qi and co‐workers developed a Ru‐based dual‐site catalyst that significantly accelerated reaction kinetics and boosts AEM water electrolysis performance [18]. Tilley and co‐workers demonstrated that precise control over the atomic‐scale distribution of Pt on Ru nanoparticles, substantially improving catalytic performance [19]. These studies highlight the catalytic advantages and potential of ruthenium‐based catalysts in alkaline HER.

Developing new ruthenium‐based catalysts is important, but understanding the underlying mechanisms is equally valuable for designing novel catalysts [9, 20]. Li and colleagues used in situ Surface‐enhanced Raman Spectroscopy (SERS) to investigate the interfacial water and reaction mechanism of ruthenium metal. They proposed that water molecules dissociate near the material surface, the produced proton transfers to the material surface with the adsorbed OH^*^ acting as a bridge [21, 22]. Koper's team suggested that in alkaline environments if a catalyst's ability to adsorb OH^*^ is ‘too weak’, water dissociation becomes the rate‐limiting step. Conversely, if the OH^*^ adsorption ability is ‘too strong’, the OH^*^ desorption step becomes the rate‐limiting step. Loading Ru onto a Pt crystal can reduce the catalyst's OH^*^ adsorption ability, significantly enhancing the catalytic performance of the catalyst [23]. Chen and colleagues found that a hydrogen‐bond network ‘gap’ exists in alkali media near the electrode surface, limiting the transfer of reactants and slowing the HER reaction rate. The Ru, which adsorbs OH^*^, enhances the continuity of the hydrogen‐bond network, thus increasing the reaction rate [24, 25]. Their further investigation confirms that surface oxophilicity conferred by Ru modulates the electric double layer, which in turn governs the alkaline HER kinetics [26].

The aforementioned studies have highlighted the crucial role of adsorbed ${\mathrm{OH}}^{*}$ species on Ru, making significant contributions to the understanding of the reaction mechanisms of ruthenium‐based catalysts in HER. Recently, Qiao and co‐workers have proposed a reconstruction mechanism of RuO_x_ under alkaline conditions during the OER [27]. However, a systematic understanding of the reconstruction phenomena of ruthenium‐based catalysts in HER is still lacking. To date, no consensus has yet been reached regarding the surface reconstruction and microscopic mechanism of the Volmer step occurring on ruthenium‐based catalysts.

In this work, high‐density nano‐needle arrays were constructed on Cu foam via a simple electrodeposition process, followed by hydrothermal loading of RuO_x_. To elucidate the catalytic mechanism of RuO_x_, in situ Raman spectroscopy and Density Functional Theory (DFT) calculations were employed. Reversible surface reconstruction under operating conditions was captured by in situ Raman spectroscopy. DFT calculations indicate that the ‘real surface’ of RuO_x_ during HER evolves into a Ru(II)O structure, which may provide a key explanation for the exceptional catalytic activity of Ru‐based materials in alkaline HER. By correlating the computational results with in situ experimental data, we propose a plausible microscopic reaction mechanism for the Volmer step. In addition, the nano‐needle array architecture significantly enhances the catalytic activity of RuO_x_, which exhibits superior performance compared with commercial Pt/C and other noble metal oxides investigated in this study. This study offers a straightforward and broadly applicable strategy, together with fundamental mechanistic insights, for improving catalyst performance, potentially paving the way toward more efficient and sustainable hydrogen production.

## 2 Results and Discussion

**FIGURE 1:**
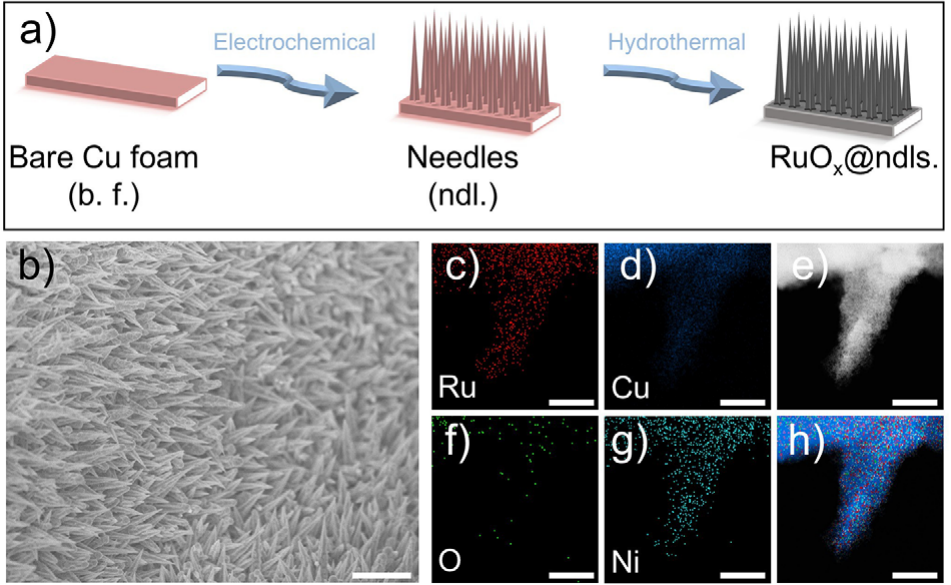
(a) Schematic illustration of the synthesis route of RuO_x_@ndl. Nano‐needle arrays were synthesized on copper foam via one‐step electrochemical deposition, followed by hydrothermal loading of ruthenium oxide onto the nano‐needles. (b) Morphology of synthesized needles imaged by scanning electron microscopy (SEM); the scale bar is 5 μm. (c–h) TEM image of RuO_x_@ndl. and the corresponding Energy Dispersive X‐ray Spectroscopy (EDS) elemental mapping of Ru, Cu, O, and Ni; the scale bar is 100 nm.

Figure 1a provides a brief overview of the sample preparation processes. The copper foam was deposited with dense nano‐needle arrays, and then it was used as catalyst supports to improve the activity of RuO_x_ and other metal oxides. As shown in Figure 1a– c, the pristine copper foam shows a relatively smooth surface. In contrast, after electrodeposition, dense nano‐needle arrays are formed on the copper foam surface (Figure 1b). They densely cover the foam with an approximate length of 3–5 μm and a density of around 1.08 × 10^8^ needles per square centimeter. Additional images from different regions are provided in the Supporting Information(Figure 1d– f). The TEM image (Figure S2) shows that the needle is well crystallized, and an interplanar spacing of 0.30 nm is observed. To obtain the elemental composition and XRD pattern of the needle‐only microstructure, the needles were deposited on carbon paper to exclude the influence of the copper foam on the analysis. EDS spectra (Table S1) show that the tip structure contains two metals, copper and nickel, and the average contents of copper and nickel are 95.6 wt.% and 4.4%, respectively. In the XRD pattern, Figure S3, there are no diffraction peaks of nickel crystals, but the picture shows that after being deposited with Ni, the sample's peaks shift to high angles, while the peaks of graphite do not change. This indicates that nickel is not deposited as an elementary substance but doped into copper crystals so that the interplanar spacing of copper becomes smaller, which makes the peak shift to a higher angle [28]. After 60 min of the hydrothermal reaction at 160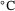, the ruthenium is successfully loaded. The sample prepared by hydrothermal synthesis at 160 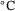 for 60 min was labeled as RuO_x_@ndl. The sample was dissolved using a 1:1 (v/v) mixed solution of 30% $H_{2}O$ 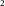 and concentrated HCl. MP‐AES measurements showed that the Ru loading was 0.178 mg ${\mathrm{cm}}^{-2}$. As shown in the TEM elemental mapping image of Figure 1c–d, the whole needle is loaded with Ru. The X‐ray diffraction of the samples in Figure S4 shows no copper peak shift or new peak related to the Ru compound. This indicates the loaded ruthenium is not intercalating or doping into needles.

The hydrothermal temperature and duration for loading RuO_x_ onto Cu foam with the nano‐needle array were systematically optimized. To evaluate the HER performance of the Ru‐based catalysts, linear sweep voltammetry measurements were performed at a scan rate of 5 mV per second in a 1 M KOH electrolyte [29]. As shown in Figure S5a, the catalyst exhibits the lowest HER overpotential at 160 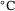 for 60 min. In addition, it shows the nearly lowest Tafel slope (Figures S5b,c and Tables S2 and S3), as well as the minimum charge‐transfer resistance among all samples(Figures S6– S9 and Tables S4 and S5). Therefore, all subsequent catalysts were synthesized under these optimized hydrothermal conditions. The Cu foam without nano‐needle arrays is denoted as b.f. (bare foam), whereas the Cu foam with nano‐needle arrays is denoted as ndl. Accordingly, RuO_x_ loaded on b.f. and ndl. are referred to as RuO_x_@b.f. and RuO_x_@ndl., respectively. Other noble metal‐loaded catalysts are denoted as RhO_x_@ndl., PtO_x_@ndl., IrO_x_@ndl., PdO_x_@ndl., AuO_x_@ndl., and AgO_x_@ndl. As shown in the EDS spectra in Figure S10, distinct signal peaks associated with the respective metals can be identified, demonstrating their successful incorporation onto the nano‐needle‐array substrate.

**FIGURE 2:**
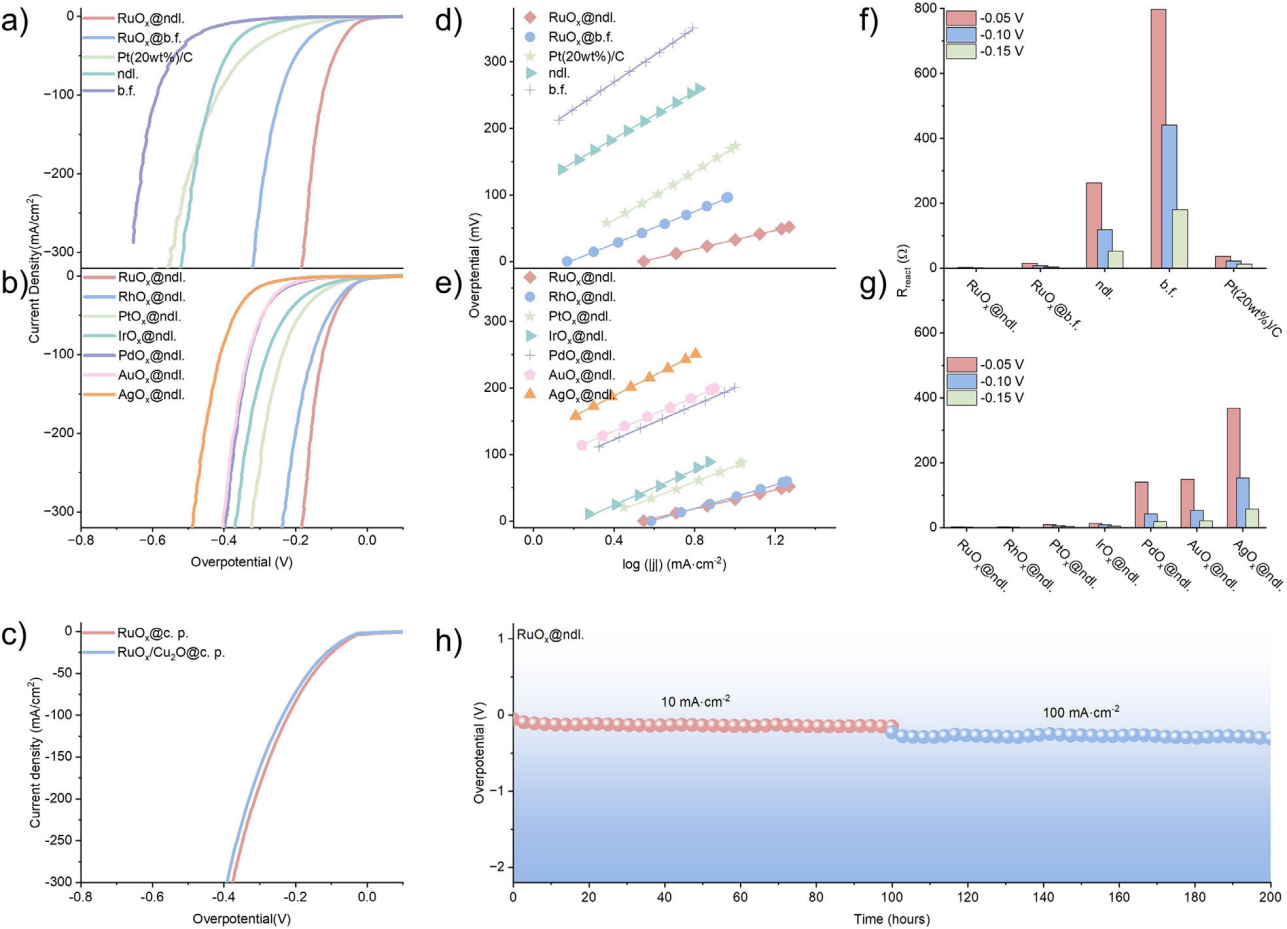
(a) Comparison of the catalytic performance of catalysts with and without nano‐needle arrays, their precursors, and commercial Pt/C, (d) corresponding Tafel slopes and (f) reaction resistance from electrochemical impedance spectra (EIS). (b) Comparison of the catalytic performance of different noble metal catalysts prepared via the same loading strategy at 160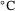 and 60 min, (e) corresponding Tafel slopes and (g) reaction resistance from EIS. (c) Comparison of the catalytic performance of RuO_x_@c. p. with RuO_x_/Cu_2_O@c. p. (h) Stability test of RuO_x_@ndl..

Figure 2a compares the HER performance of b.f., ndl., RuO_x_@ndl., RuO_x_@b.f., and commercial Pt/C. The Cu foam with nano‐needle array (ndl.) exhibits enhanced catalytic activity compared with the bare foam (b.f.). Notably, RuO_x_@ndl. outperforms RuO_x_@b.f., indicating that the nano‐needle architecture significantly promotes the activity of RuO_x_. At 10 mA·cm 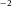, the nano‐needle arrays lower the overpotential of RuO_x_ from 101 to 32 mV. Commercial Pt/C was loaded on Cu foam using Nafion as a binder with a loading mass of 0.5 mg·cm 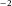. Both RuO_x_@ndl. and RuO_x_@b.f. exhibit substantially higher activity than Pt/C (174 mV at 10 mA·cm 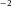), underscoring the superior performance of RuO_x_ in alkaline HER. Subsequently, the comparison of Tafel slopes in Figure 2d shows that RuO_x_@b.f. exhibits a Tafel slope of 121 mV·dec 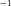, which is 70.4% higher than that of RuO_x_@ndl.(71 mV·dec 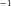; Table S6). In contrast, Pt/C supported on Cu foam (182 mV·dec 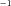) shows a much higher Tafel slope than RuO_x_ loaded on Cu foam. The Tafel slopes of RuO_x_@ndl. are close to 80 mV·dec 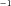, indicating that the hydrogen evolution reaction catalyzed by RuO_x_@ndl. is governed by a Volmer–Heyrovsky mechanism, with mixed control from the Volmer and Heyrovsky steps [30, 31]. Furthermore, the electrochemical impedance spectra in Figure S11 and the charge‐transfer resistance comparison of Figure 2f reveal that RuO_x_@ndl. exhibits lower charge‐transfer resistance (R_react_) than RuO_x_@b.f. and Pt/C at applied potentials of 0.05, 0.10, and 0.15 V (vs. RHE), further highlighting the critical role of the nano‐needle architecture in promoting HER kinetics. Notably, both RuO_x_@ndl. and RuO_x_@b.f. display smaller R_react_ than Pt/C, underscoring the superior catalytic performance of RuO_x_ in alkaline HER. As presented in Figure S12, the nanoneedle synthesis procedure was employed, with the sole variation being the pH of the electrodeposition process. This yielded a relatively flat Cu foam with surface Ni incorporation and without nanoneedle arrays, hereafter referred to as f.f. (flat foam). The overpotential at 10 mA·cm 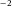 and the charge‐transfer resistance in Figures S12 and S13 both decrease in the order of RuO_x_@b. f. > RuO_x_@f. f. > RuO_x_@ndl., while the Tafel slope of RuO_x_@b. f. remains higher than those of RuO_x_@f. f. and RuO_x_@ndl. These observations demonstrate that both the incorporation of Ni species and the needle‐like morphology play beneficial roles in enhancing HER performance. In addition, because very weak Cu_2_O peaks were observed in Figure S3 and the influence of Cu_2_O on the catalytic performance of RuO_x_ remained uncertain, Cu_2_O nanoparticles were separately synthesized for a more rigorous evaluation. Their effect on the performance of RuO_x_ was investigated by mixing Cu_2_O with RuO_x_ and loading the mixture onto carbon paper. As shown in Figure 2c, RuO_x_/Cu_2_O@c. p. exhibits nearly identical performance to RuO_x_@c. p., with overpotentials of −391 and −376 mV at 300 mA ${\mathrm{cm}}^{-2}$, respectively. It can be seen from Figure S15 that the charge‐transfer resistance of RuO_x_/Cu_2_O@c. p. is slightly higher than that of RuO_x_@c. p. And the two samples exhibit similar Tafel slopes in Figure S14. Considering these experimental results together with the fact that Cu_2_O can be reduced to metallic Cu during the HER process, it can be concluded that the Cu_2_O formed during synthesis has little influence on the catalytic performance of RuO_x_. As shown in Figure 2b, among the catalysts loaded with different metals on the nano‐needle arrays, RuO_x_ exhibits the best catalytic performance. The activity follows the order: RuO_x_ (32 mV at 10 mA·cm 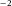) > RhO_x_ (36 mV at 10 mA·cm 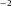) > PtO_x_ (83 mV at 10 mA·cm 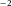) > IrO_x_ (108 mV at 10 mA·cm 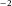) > PdO_x_ (201 mV at 10 mA·cm 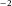) > AuO_x_ (213 mV at 10 mA·cm 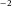) > AgO_x_ (282 mV at 10 mA·cm 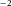). Among these metal oxide catalysts, PtO_x_, IrO_x_, PdO_x_, and AuO_x_ exhibit comparable Tafel slopes of approximately 120 mV·dec 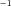 (Figure 2e and Table S7). A Tafel slope close to 120 mV·dec 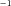 indicates that the Volmer step, involving water dissociation, is likely the rate‐determining step. Moreover, the trend of charge‐transfer resistance shown in Figure 2g and Figure S16 is consistent with the catalytic activity ranking observed in the LSV curves. Specifically, PdO_x_@ndl., AuO_x_@ndl., and AgO_x_@ndl. exhibit significantly higher R_react_ than RuO_x_. Notably, RuO_x_ displays the lowest charge‐transfer resistance among all metal oxides at applied potentials of −0.10 and −0.15 V (vs. RHE). Moreover, the stability tests shown in Figure 2h reveal that RuO_x_@ndl. operates continuously for 200 h at current densities of 10 and 100 mA·cm 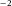 without noticeable performance degradation, demonstrating its excellent long‐term stability.

**FIGURE 3:**
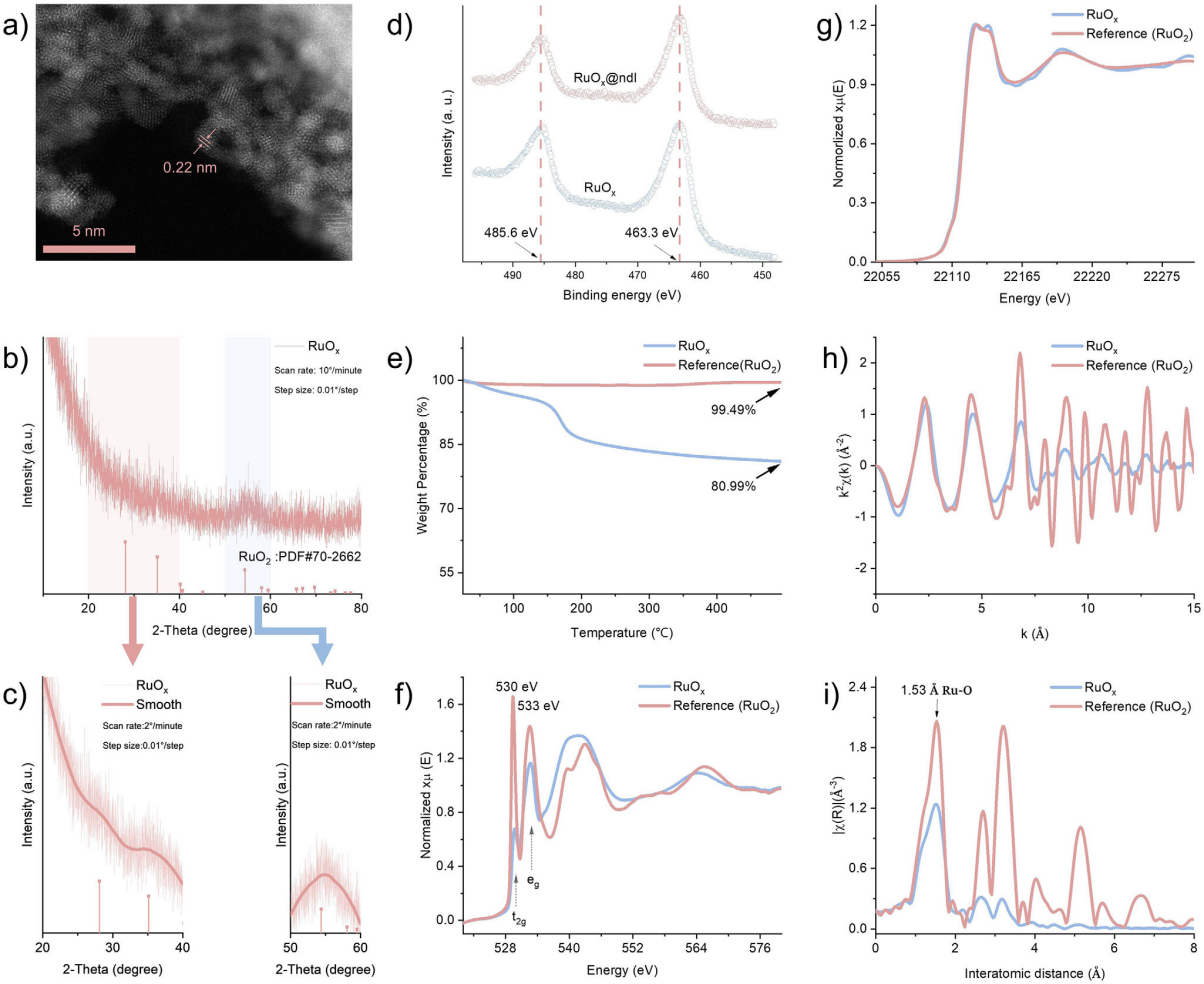
(a) High‐angle annular dark‐field imaging (HAADF) picture of RuO_x_. (b) XRD pattern of RuO_x_ recorded at 10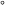 $\min^{-1}$. (c) XRD patterns recorded at 2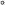 $\min^{-1}$, with the smoothed curves obtained using the Savitzky–Golay method (window points: 700). (d) High‐resolution Ru 3p X‐ray Photoelectron Spectroscopy(XPS) spectra of RuO_x_ and RuO_x_@ndl. (e)Thermogravimetric analysis (TGA) of the as‐synthesized RuO_x_ and commercial RuO_2_ under air atmosphere. (f) Oxygen's Near‐Edge X‐ray Absorption Fine Structure (NEXAFS) spectra of RuO_x_ and RuO_2_; (g) Normalized Ru K‐edge XANES spectra of RuO_x_ and RuO_2_, and corresponding (h) $k^{2}$‐weighted EXAFS spectra; (i) FT‐EXAFS spectra.

As discussed above, RuO_x_ exhibits outstanding catalytic activity for the hydrogen evolution reaction in alkaline media, which makes it particularly important to further investigate its microscopic structure and reaction mechanism. To further confirm the structure of the RuO_x_, RuO_x_ was synthesized without the substrate (Cu foam) via the same synthetic procedure. From Figure 3a, the HAADF image of RuO_x_ clearly shows that the Ru atoms are well arranged with noticeable lattice planes with interplanar spacing of 0.22 nm (Additional STEM images of RuO_x_ are shown in Figure S17). As shown in Figure 3b, the XRD pattern of RuO_x_ is presented. The characteristic diffraction peaks corresponding to RuO_2_ at the (1 1 0), (1 0 1), and (2 1 1) planes can be identified. To further enhance the resolution and distinguishability of these three peaks, the XRD data in the 2θ ranges of 20 to 40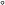 and 50 to 60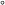 were re‐collected using a slower scanning rate of 2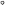 $\min^{-1}$ (Figure 3c). After applying Savitzky‐Golay smoothing to remove background noise, the diffraction peaks of RuO_2_ at (1 1 0), (1 0 1), and (2 1 1) became more pronounced. These results are consistent with the STEM observations, indicating that RuO_x_ consists of ultrafine nanocrystals with an average size of approximately 2 nm. As revealed by the Ru 3p and Ru 3d XPS spectra in Figure 3d and Figure S18b, RuO_x_ and RuO_x_@ndl. exhibit very similar spectral features. In the XPS survey spectrum of RuO_x_ (Figure S18c), only Ru and O signals are detected (Ru: 38.95%; O: 61.05%), with no Cl signal observed. Combined with the thermogravimetric analysis (TGA, Figure 3e), which shows a weight loss of 19%, it can be inferred that RuO_x_ contains crystalline water. Furthermore, XRD analysis of the residual solid after TGA confirms that the remaining phase is RuO_2_ (Figure S19), further verifying that the synthesized material is hydrated ruthenium oxide rather than metallic Ru.

The synthesized RuO_x_ structure was further investigated by O K‐edge near‐edge X‐ray absorption fine structure (NEXAFS) and Ru K‐edge X‐ray absorption spectroscopy (XAS). Considering that the surface atomic structure of RuO_x_ nanoparticles may differ from their bulk structure, soft X‐ray absorption spectroscopy was employed to probe the surface and near‐surface regions. As shown in Figure 3f, the O K‐edge NEXAFS spectrum exhibits two pre‐edge features at approximately 530 and 533 eV, which can be assigned to transitions from O 1s to unoccupied O 2p states hybridized with Ru 4d $t_{2g}$ and $e_{g}$ orbitals, respectively [32, 33]. It has been reported that the weakening of the $t_{2g}$ peak is indicative of under‐coordination of surface metal atoms [33, 34]. Accordingly, Ru atoms on the surface of RuO_x_ are coordinatively unsaturated compared to those in RuO_2_.

To further elucidate the structural characteristics of RuO_x_, RuO_2_ with a rutile structure (space group $P4_{2}/mnm$) was used as a reference. As depicted in Figure 3g, the Ru K‐edge XANES spectra of RuO_2_ and RuO_x_ nearly overlap, suggesting that the bulk Ru atoms in RuO_x_ possess a similar oxidation state to those in RuO_2_. The corresponding extended X‐ray absorption fine structure (EXAFS) spectrum (Figure 3h) exhibits a high signal‐to‐noise ratio; however, when the k value exceeds 8 Å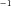, the oscillation amplitude of $k^{2}\chi \left(k\right)$ decreases markedly, indicating poor long‐range order, which can be attributed to the small particle size and the high fraction of surface atoms. In the R‐space EXAFS spectra (Figure 3i), both RuO_x_ and RuO_2_ exhibit a comparable peak at approximately 1.52 Å, which can be assigned to the Ru–O scattering path [35, 36]. Notably, the first coordination shell of RuO_x_ is consistent with that of RuO_2_, whereas the higher‐shell features are significantly attenuated. These results collectively demonstrate that although the surface Ru atoms in RuO_x_ are under‐coordinated, the bulk phase of RuO_x_ retains a RuO_2_‐like structure.

**FIGURE 4:**
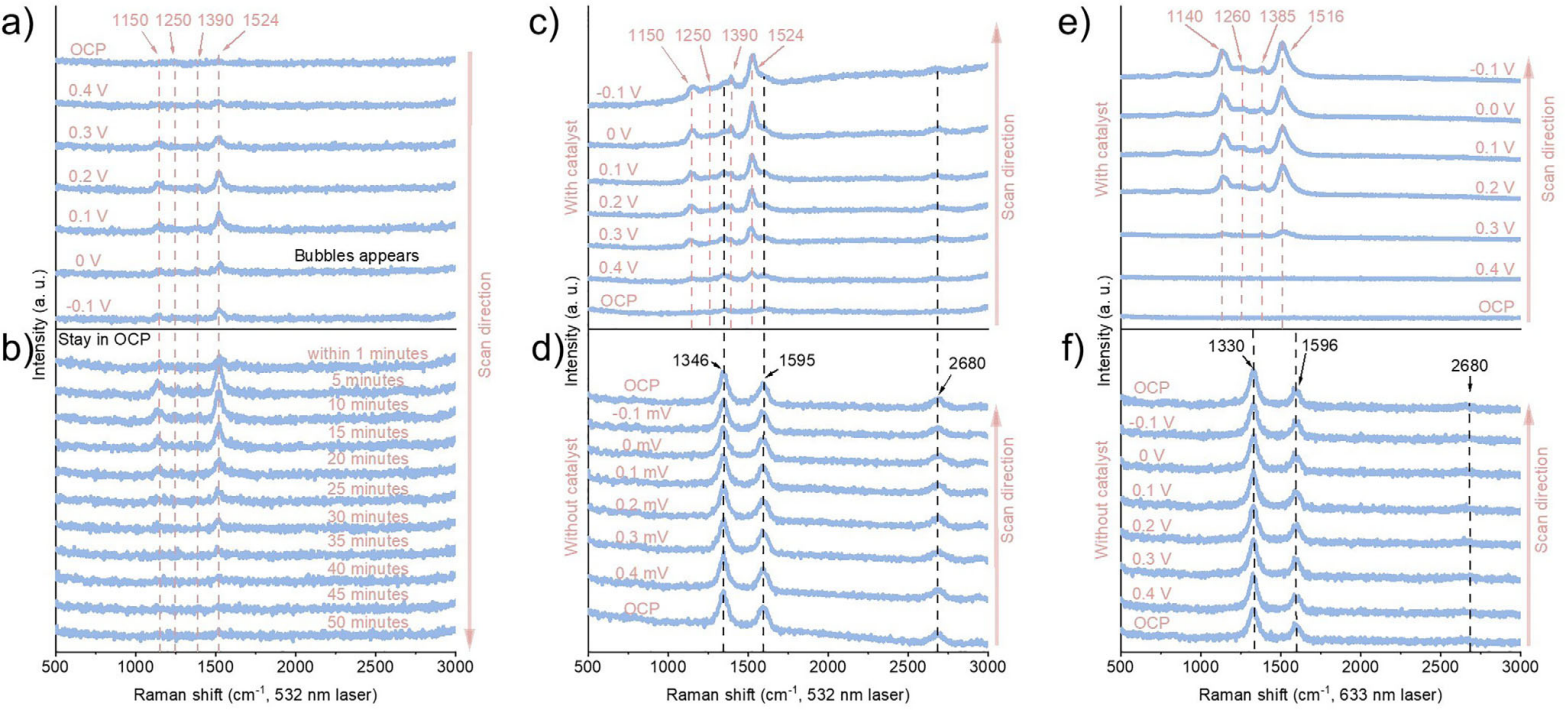
(a) In situ Raman spectra of RuO_x_ in 1 M KOH using a 532 nm laser. (b) Temporal evolution of the Raman signal after removing the applied potential. (c) Differential response of the catalyst and glassy carbon substrate signals under applied voltage using a 532 nm laser. (d) Glassy carbon substrate signals under the same conditions as (c) but without the catalyst. (e) Raman spectra of RuO_x_ using a 633 nm laser. (f) Corresponding glassy carbon substrate signals without the catalyst.

Surface restructuring plays a pivotal role in the predictive design of catalysts [37]. To study the reaction mechanism and reconstruction of RuO_x_, in situ Raman spectroscopy was used to observe surface changes during the reaction process of RuO_x_. The experimental results are shown in Figure 4a. The scan was initiated from the open‐circuit potential (OCP). As shown in Figure S20, the OCP of the RuO_x_‐loaded working electrode measured in the Raman cell was approximately 0.95 V (RHE). When the voltage is higher than 0.4 V (RHE), no absorption peaks appear, and the absorption peaks of RuO_2_ E_g_, A_1g_, and B_2g_ located between 500–750 cm^−1^ also do not appear. This indicates that although the internal atomic arrangement of the synthesized RuO_x_ is consistent with that of RuO_2_, the surface structures are different. When the voltage is reduced to below 0.4V (RHE), four reversible Raman signals slowly appear with wavenumbers of 1150, 1250, 1390, and 1524 cm^−1^. Figure 4b shows that when the applied potential is removed, the peaks do not immediately disappear; instead, the peak intensities grow slowly within 10 min and then slowly disappear within 50 min. Before establishing the entire reaction mechanism, two key issues must first be clarified: (i) whether the signal arises from the catalyst itself or from the glassy carbon electrode, and (ii) whether the observed signal originates from Raman scattering or fluorescence.

To confirm the reliability and origin of the Raman signal, the experiment was repeated at regions of the electrode with thin catalyst coverage, where both RuO_x_ and glassy carbon signals were simultaneously observed (1346 ${\mathrm{cm}}^{-1}$ for the D band of glassy carbon and 1595 ${\mathrm{cm}}^{-1}$ for the G band of glassy carbon, Figure 4c). Notably, the glassy carbon signals remain largely unchanged with the changes in the applied potential, whereas the RuO_x_ signal increases obviously as the potential decreases. In the control experiment without catalyst (Figure 4d), only the glassy carbon signals were detected, showing negligible variation with potential change. These observations unambiguously confirm that the potential‐dependent signal originates from the surface of RuO_x_.

To find out if these peaks are Raman scattering or fluorescence signals, another laser with a wavelength of 633 nm was used in a repeat test. As shown in Figure 4e, these four peaks still appear when using a 633 nm laser, and their positions only shift slightly (within 10 cm^−1^). This indicates that these peaks are Raman signals and not fluorescence, as the latter are broader and disappear or shift significantly when a longer wavelength laser is used. Similarly, in the control experiment conducted without RuO_x_, the following observations were also recorded, as shown in Figure 4f. Two peaks at 1330 and 1596 cm^−1^ are observed. They correspond to the D and G peaks of the glassy carbon electrode, which do not change with potential.

Therefore, the appearance of the peaks can be explained by the following reaction mechanism. When a potential below 0.4 V is applied to RuO_x_, its surface undergoes reversible reconstruction, and the reconstructed surface adsorbs species such as hydrogen, water, and hydroxyl groups, giving rise to the observed signal. As the potential decreases, the amount of adsorbed species increases, leading to an enhancement of the Raman intensity. When the potential returns to the OCP, in the absence of overpotential, the adsorbed species desorb and the surface reconstruction reverses.

It is essential to determine the true surface structure of RuO_x_ during the reaction for identifying the actual reaction sites and understanding the origin of the superior performance of Ru‐based catalysts in alkaline environments. DFT calculations were performed to obtain vibrational frequencies, which were then compared with experimental data to validate the reconstructed surface structure. RuO_2_, with under‐coordinated surface Ru atoms, was chosen as a model compound. This choice is supported by several observations: EXAFS analysis confirmed that RuO_x_ shares the same internal atomic arrangement as RuO_2_, and STEM imaging revealed well‐defined lattice fringes, indicating that the bulk structure of RuO_x_ is analogous to RuO_2_. Meanwhile, O K‐edge NEXAFS and XPS results show that the surface Ru atoms of RuO_x_ are under‐coordinated. Collectively, these findings validate the use of RuO_2_ with under‐coordinated surface Ru atoms as a reaction model.

In the following, the (1 1 0) facet of RuO_2_ with under‐coordinated surfacial Ru atoms was modelled, and its possible reconstructed structure was modelled as well. The predicted reconstruction undergoes adsorption of new‐formed hydrogen atom (H^*^) onto the oxygen site of RuO_x_(Equation 1). When the concentration of H^*^ is too high, the hydroxyl group on the surface becomes unstable and is expel from the surface, Equation 2 [38, 39, 40, 41]. This is why RuO_x_ has high pseudo‐capacitance [42, 43].

(1) $${\mathrm{RuO}}_{x}+n\;H_{2}O+n\;e^{-}\to {\mathrm{RuO}}_{\left(x-\frac{n}{2}\right)}{\left(\mathrm{OH}\right)}_{n}+n\;{\mathrm{OH}}^{-}$$

(2) $${\mathrm{RuO}}_{\left(2-\frac{n}{2}\right)}{\left(\mathrm{OH}\right)}_{n}+n\;e^{-}\to {\mathrm{RuO}}_{\left(2-n\right)}+n\;{\mathrm{OH}}^{-}$$

**FIGURE 5:**
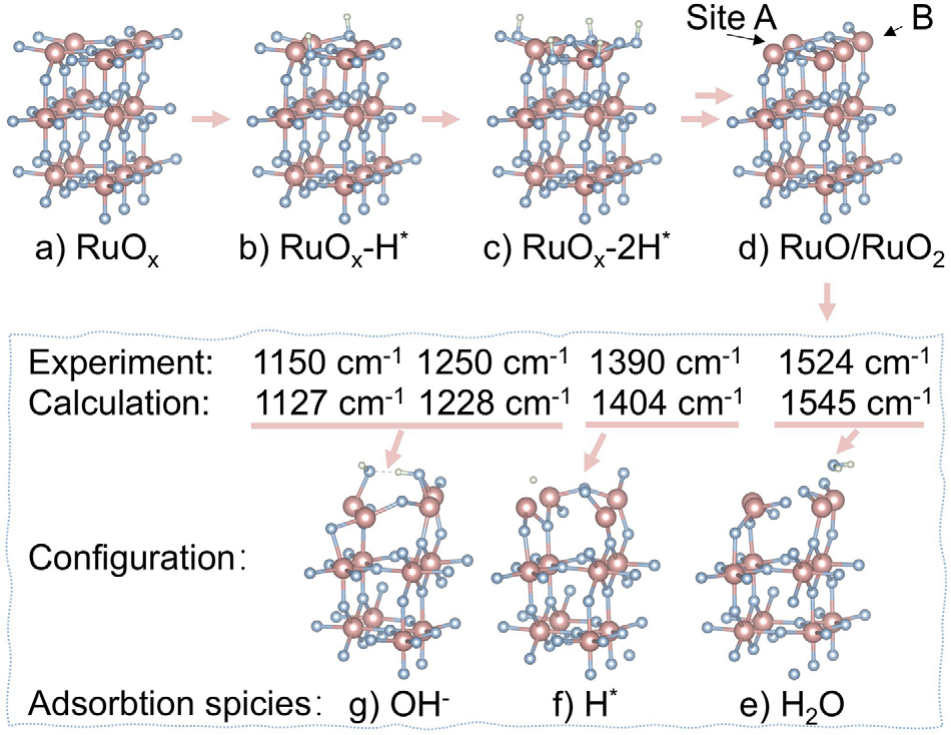
DFT calculations of the reconstructed structures and comparison with Raman signals. (a) Optimized RuO_x_ structure. (b) H^*^ adsorption. (c) Further H^*^ adsorption. (d) Surficial oxygen removal, with the surface layer reconstructed to RuO while the bulk phase remains RuO_2_. (e–g) Adsorption of water, H^*^, or hydroxide on the reconstructed RuO/RuO_2_ surfaces.

The reconstructed ${\mathrm{RuO}}_{\left(2-n\right)}$ surface represents the true active surface structure of the catalyst during HER. After exploring numerous structural models, it was found that setting n=1 yields a reasonable theoretical model, and the calculated results are in good agreement with the experimental observations. Assuming n = 1, as shown in Figure 5d, the ruthenium oxide with the surface of Ru(II)O and the bulk of Ru(IV)O_2_ (RuO/RuO_2_) was modelled and optimized. Then, after optimizing the adsorption models of H_2_O, H^*^ and OH^−^, the structures (e), (f), and (g) were obtained, as well as their vibrational frequencies. The calculation data match the experimental results as shown in Figure 5. The calculated wavenumber of 1545 cm^−1^ arises from the scissoring vibration of adsorbed H_2_O (Figure 5e), which corresponds to the experimental data of 1524 cm^−1^. The H^*^ is adsorbed at the bridge site of two Ru atoms in Figure 5f, and the vibration of H^*^ in the vertical direction is calculated to be 1404 cm^−1^, which corresponds to the experimental data of 1390 cm^−1^. In Figure 5g, cleaved water molecules at site A resulted in two hydroxyl groups, and their synergistic vibration bending resulted in two components at 1228 and 1127 cm^−1^, which are close to the experimental data of 1250 and 1150 cm^−1^. A video showing how these atoms vibrate was uploaded as supporting information. So, by comparing the experimental data and DFT results, it can be confirmed that the RuO_x_ was reconstructed to RuO/RuO_2_ structure.

**FIGURE 6:**
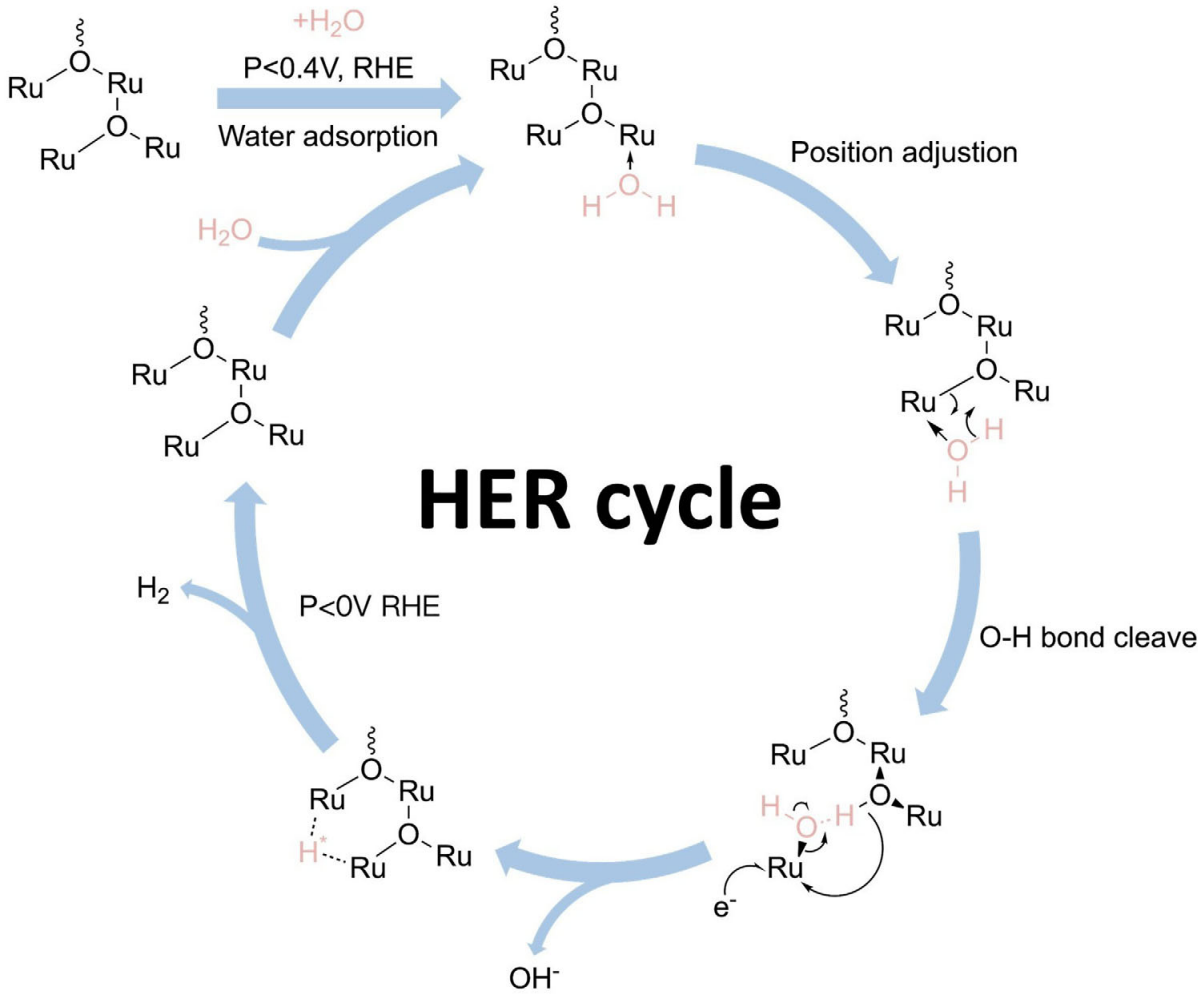
Scheme of the proposed Volmer step reaction mechanism on Ru(II)O.

By combining previous research [38, 39, 40, 41] and the current study, a detailed reaction mechanism can be assumed as follows. The RuO_x_ first adsorbs H^*^ when the potential decreases. With more H^*^ adsorbed, the surficial oxygens are removed at about 0.4V (RHE, 1M KOH), and the RuO_x_ is reconstructed to RuO/RuO_2_ structure, followed by adsorption with OH^−^, H^*^ and H_2_O. The actual catalyst for the HER is Ru(II)O species. The supposed Volmer step is shown in a 2D figure in Figure 6 and underwent the following steps: (1) when the potential is below 0.4V RHE, the reconstruction formed RuO on the surface of RuO_x_ and a water molecule is adsorbed on site B (the 3D configuration as in Figure 5e); (2) the adsorbed water is transferred to site A and then cleaves to form two hydroxyl groups (forming the configuration of Figure 5g); (3) one of the OH^*^ accepts an electron and then leaves the surface as OH^−^ group, the other is rearranged to form adsorbed H^*^ (the 3D configuration as Figure 5f). As the overpotential decreases below 0 V (RHE), the hydrogen atoms adsorbed between Ru atoms desorb in the form of H_2_, and the surface reaction subsequently enters the next catalytic cycle.

## 3 Conclusion

Ruthenium oxide exhibits outstanding HER activity in alkaline media, yet its true surface structure and reaction mechanism under operating conditions remain elusive. Herein, in situ Raman spectroscopy reveals that RuO_x_ undergoes a reversible surface reconstruction during HER. DFT calculations confirm the formation of metastable Ru(II)O species on the surface. Based on these results, a Volmer reaction pathway on reconstructed RuO_x_ is proposed, highlighting the critical role of surface reconstruction in promoting water dissociation and hydrogen adsorption.

Furthermore, a series of precious metal oxides were loaded onto copper foam with nano‐needle arrays for systematic comparison. At −0.15 V (vs. RHE), RuO_x_ delivers a current density that is 1.8–119.5 times higher than those of RhO_x_, PtO_x_, IrO_x_, PdO_x_, AuO_x_, and AgO_x_, demonstrating its exceptional alkaline HER activity. The nano‐needle arrays markedly enhance the catalytic performance of RuO_x_, resulting in an approximately 6.5‐fold increase in current density at −0.15 V (vs. RHE). The RuO_x_@ndl. catalyst also exhibits excellent long‐term stability, indicating strong potential for practical applications. Overall, this work establishes an efficient and stable alkaline HER catalyst and provides direct mechanistic insight into the reversible surface reconstruction of ruthenium oxide, offering guidance for the rational design of advanced Ru‐based catalysts.

## 4 Experimental

### 4.1 Materials

Nickel(II) sulfate hexahydrate was purchased from BDH Chemicals Ltd, polyethylene glycol‐600 was obtained from Alfa Aesar, Rhodium(III) chloride trihydrate, sodium hexachloroplatinate(IV) hexahydrate, sodium hexachloroiridate hydrate, sodium tetrachloropalladate (II), sodium tetrachloroaurate (III), silver sulfate, and platinum on carbon (20 wt.%) were purchased from Macklin, other chemicals without specific instructions were acquired from Sigma–Aldrich and used without further purification. The working electrode was fabricated using copper foam with a thickness of 1mm. The foam possessed a nominal pore density of 130 pores per inch and was purchased from Suzhou Zhengtairong Scientific Research New Materials Ltd.

### 4.2 Needle Synthesis

The needle synthesis followed a reported method [28, 44]. Briefly, copper foams were successively washed with $0.5\;M\;H_{2}{\mathrm{SO}}_{4}$, acetone, ethanol, and deionized water under ultrasonication. A three‐electrode system was employed for electrodeposition with Ag/AgCl (sat. KCl) reference and a Pt plate counter electrode. The electrolyte composition was: ${\mathrm{CuSO}}_{4}$ (0.03 M), ${\mathrm{NiSO}}_{4}$ (0.0024 M), ${\mathrm{NaH}}_{2}{\mathrm{PO}}_{2}$ (0.24 M), ${\mathrm{Na}}_{3}C_{6}H_{5}O_{7}$ (0.05 M), $H_{3}{\mathrm{BO}}_{3}$ (0.5 M), and polyethylene glycol (6 g·L 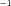). The deposition was carried out at pH = 8 with a constant potential of −1.17V (vs. Ag/AgCl(sat. KCl)) for 20min at 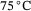.

### 4.3 Electrode Preparation

The substrates with needles were loaded with Ru via hydrothermal reaction using 10 mL of 1 mg·mL ^−1^ ${\mathrm{RuCl}}_{3}\cdot {\mathrm{xH}}_{2}O$ solution as the Ru source. The reaction was conducted at 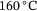 for 10, 20, 40, 60, or 120min. The obtained samples were designated as RuO_x_@ndl._10min, RuO_x_@ndl._20min, RuO_x_@ndl._40min, RuO_x_@ndl._60min, and RuO_x_@ndl._120min, respectively. For temperature optimization, the catalysts were synthesized under identical reaction conditions with a fixed reaction time of 60 min, while the hydrothermal temperature was varied at 40, 80, 100, 120, 160, and 200 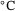. The resulting samples were denoted as RuO_x_@ndl._40 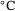, RuO_x_@ndl._80 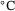, RuO_x_@ndl._100 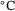, RuO_x_@ndl._120 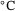, RuO_x_@ndl._160 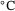, and RuO_x_@ndl._200 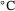, respectively.

RuO_x_@b. f. and RuO_x_@f. f. were employed as control samples for RuO_x_@ndl._60min. Specifically, b. f. (bare foam) represents pretreated Cu foam without electrochemical deposition, while f. f. (flat foam) refers to the Cu foam prepared without altering the pH during electrodeposition, yielding a relatively flat surface morphology without nanoneedle arrays. The preparation of Cu_2_O was carried out according to a previously reported method [45]. Briefly, 27 mL of deionized water, 0.3 mL of 0.1 M CuCl_2_ solution, 1.95 mL of 0.2 M ${\mathrm{NH}}_{2}\mathrm{OH}\cdot \mathrm{HCl}$, and 0.75 mL of 1 M KOH were mixed in a beaker under stirring. The mixture was then aged overnight, followed by centrifugation and washing twice. The resulting solid was redispersed in 2 mL of deionized water. Subsequently, 0.1 mL of the above Cu_2_O dispersion was mixed with 0.15 mL of RuO_x_ dispersion (1.17 ${\mathrm{mg}}_{\mathrm{Ru}}$ ·mL 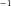) and 0.1 mL of diluted Nafion solution (deionized water:ethanol:Nafion solution =19:5:1). After thorough mixing, the obtained ink was drop‐cast onto hydrophilic carbon paper (c. p.) in three successive aliquots under an infrared lamp, followed by drying to obtain RuO_x_/Cu_2_O@c. p. RuO_x_@c. p. was prepared using the same procedure, except that no Cu_2_O nanoparticles were added. The other metal oxides (MO_x_) were synthesized via hydrothermal reaction at 160 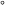 C for 60 min using Rhodium chloride trihydrate, sodium hexachloroplatinate hexahydrate, sodium hexachloroiridate hydrate, sodium tetrachloropalladate, sodium tetrachloroaurate, and silver sulfate as the metal sources, with a concentration of 1 mg·mL ^−1^. The synthesized catalysts were denoted as RuO_x_@ndl., RhO_x_@ndl., PtO_x_@ndl., IrO_x_@ndl., PdO_x_@ndl., AuO_x_@ndl., and AgO_x_@ndl.

### 4.4 Characterization Details

The structure and morphology of the samples were investigated using scanning electron microscopy (SEM, JEOL 7600 and 6701). Phase composition and chemical states were characterized by X‐ray diffraction (XRD; STOE SEIFERT diffractometer and Rigaku SmartLab diffractometer with Cu Kα radiation) and X‐ray photoelectron spectroscopy (XPS; Thermo Scientific K‐Alpha photoelectron spectrometer). The Ru 3d and Ru 3p spectra of RuO_x_ were collected on a gold‐coated single‐crystal silicon wafer to minimize interference from the C 1s signal of the conductive carbon tape. In contrast, the XPS survey spectrum of RuO_x_ was recorded on conductive carbon tape to avoid interference from the Au signal.

Near‐edge X‐ray absorption fine structure (NEXAFS) experiments were performed at the B07‐B beamline of Diamond Light Source (UK) during a commissioning beamtime. O K‐edge NEXAFS measurements were conducted in total electron yield (TEY) mode at the ES‐2 end station for Ambient Pressure NEXAFS. Details of the O K‐edge NEXAFS measurements are as follows. The catalyst powder and reference standard materials were dispersed on gold‐coated silicon chips, which ensured good conductivity and prevented sample contamination. The samples were illuminated by an incident beam sourced from a bending magnet and plane grating monochromator (PGM) with a spot size of approx. 200μm×200μm. The pressure in the specimen chamber was controlled at $1\times 10^{-7}$ mbar. The ruthenium loading amount of RuO_x_@ndl. was determined as follows. The 1 cm^2^ sample was dissolved in 50 mL HCl (36%) and H_2_O_2_ (30%) solution with a (v/v) ratio of 1:1 until it was completely dissolved. The solution was then diluted before the Ru concentration was determined by MP‐AES (Agilent 4210 MP‐AES).

The synthesized ruthenium oxide (prepared via the same hydrothermal method) was used for in situ Raman spectroscopy measurements. The electrochemical cell was equipped with a glassy carbon working electrode, Pt wire counter electrode, and Ag/AgCl (sat. KCl) reference electrode. Spectra were recorded with 16 accumulations and 1s exposure time per accumulation.

Geometry optimization and vibrational frequency calculations of RuO_x_ were performed using density functional theory (DFT) via the Vienna Ab initio Simulation Package (VASP) [46, 47, 48, 49]. The projector augmented wave (PAW) method was employed with the Perdew–Burke–Ernzerhof (PBE) generalized‐gradient approximation (GGA) functional [50, 51, 52]. Calculations utilized an energy cutoff of 400eV and a Monkhorst–Pack *k*‐point sampling of 3×3×1 [53]. A first‐order Methfessel–Paxton smearing with σ=0.25eV was applied to account for electronic states near the Fermi level [54]. A 15Å vacuum layer was incorporated to model surface species and mitigate periodic image interactions. The RuO_x_ surface, modeled as 2×2 surface unit cells, was investigated. During optimization, the top three layers were allowed to relax while the bottom two layers remained fixed. The lattice constant was optimized prior to surface model construction. Structural visualizations were generated using VESTA software [55].

### 4.5 Electrochemical Tests

All electrochemical measurements were performed in a three‐electrode system controlled by a potentiostat. 1MKOH served as the electrolyte. Except for the stability tests conducted in an H‐type electrolytic cell to prevent gas crossover between the electrodes, all other electrochemical measurements were performed using a simple electrochemical cell. The working electrode was fabricated with a geometric surface area of $1\;\;cm^{2}$. Commercial Pt/C (20 wt%) was dispersed in a solution containing 5 wt.% Nafion as a binder to prepare an ink with a concentration of 5 mg·mL 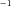. An aliquot of 100 μL of the ink was drop‐cast onto Cu foam, corresponding to a catalyst loading of 0.5 mg·cm 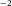, for electrochemical measurements. All potentials were measured against an Hg/HgO (1M KOH) reference electrode and converted to the reversible hydrogen electrode (RHE) scale according to the equation:

(3) $$E_{\text{RHE}}=E_{\text{Hg/HgO}}+E_{\text{Hg/HgO}}^{0}+0.059\times \text{pH}$$

The electrochemical measurements were conducted using a CHI 660E electrochemical workstation (CH Instruments, Shanghai, China) at room temperature.

## Conflicts of Interest

The authors declare no conflicts of interest.

## Supporting information


**Supporting File**: supinfo/advs76132‐sup‐0001‐SuppMat.pdf.

**Supplemental Movie**: supinfo/advs76132‐sup‐0002‐MovieS1.mov.

## Data Availability

The data that support the findings of this study are available from the corresponding author upon reasonable request.
