# Supplementary material for: Study on the Reaction Mechanism of RuOx and Its Enhanced Hydrogen Evolution Reaction Activity
Source: Adv Sci (Weinh). 2026 Jun 22:e76132. Online ahead of print. doi: 10.1002/advs.76132 (PMC13336886; doi:10.1002/advs.76132)
Supplement: Supplementary file 1 — Supporting File: supinfo/advs76132‐sup‐0001‐SuppMat.pdf. [file ADVS-9999-e76132-s001.pdf]

Study on the Reaction Mechanism of RuO_x and its Enhanced Hydrogen Evolution Reaction Activity

Liquan Zhang,+,[a],[b] Haobo Dong,+,[c] Jianrui Feng,+,[a] Longxiang Liu,[d] Fei Guo,[a] Liqun Kang,[e] Guoliang Chai,[f] Mohamed A. Ghanem,[g] Xinde Chen,*[b] Ivan P. Parkin*[a]

Contents

1. Supporting Tables 2

2. Supporting Figures 10

[a] Dr. L. Zhang, J. Feng, F. Guo, Prof. Dr. I. P. Parkin*
Department of Chemistry, Faculty of Mathematical and Physical Sciences, University College London, London WC1H 0AJ, UK
E-mail: i.p.parkin@ucl.ac.uk

[b] Dr. L. Zhang, Prof. X. Chen*
Guangzhou Institute of Energy Conversion, Chinese Academy of Sciences, No.2 Nengyuan Road, Tianhe District, Guangzhou 510640, PR China E-mail: chenxd@ms.giec.ac.cn

[c] Prof. H. Dong
School of Future Technology, South China University of Technology, Guangzhou, 511442, China

[d] Dr. L. Liu
Department of Materials, University of Oxford, Oxford, OX1 3PH, UK

[e] Dr. L. Kang
Max-Planck-Institut für Chemische Energiekonversion, 45470 Mülheim an der Ruhr, Deutschland

[f] Dr. G. Chai
State Key Laboratory of Structural Chemistry, Fujian Institute of Research on the Structure of Matter, Chinese Academy of Sciences (CAS)

[g] Prof. M. A. Ghanem
Department of Chemistry, College of Sciences, King Saud University, P.O. Box 2455, Riyadh 11451, Saudi Arabia

[+] These authors contributed equally.

1. Supporting Tables

List of Tables

S1. EDS spectrum analysis of needles, the needles are deposited on carbon paper with the same method. 3
S2. Tafel slopes of catalysts with different hydrothermal reaction time 4
S3. Tafel slopes of catalysts with different hydrothermal reaction temperature 5
S4. Electrochemical impedance spectroscopy (EIS) of catalysts with different hydrothermal reaction time. 6
S5. EIS of catalysts with different hydrothermal reaction temperature. 7
S6. Tafel slopes of different catalysts 8
S7. Tafel slopes of different metals 9

Table S1. EDS spectrum analysis of needles, the needles are deposited on carbon paper with the same method.

Cu ndl.	Cu (wt%)	Ni (wt%)
Site 1	95.7	4.3
Site 2	94.4	5.6
Site 3	96.6	3.4
Average percentage	95.6	4.4

Table S2. Tafel slopes of catalysts with different hydrothermal reaction time

Samples	Tafel slope ($mV \cdot dec^{-1}$)
RuO_x@ndls._10min	94
RuO_x@ndls._20min	88
RuO_x@ndls._40min	70
RuO_x@ndls._60min	71
RuO_x@ndls._120min	139

Table S3. Tafel slopes of catalysts with different hydrothermal reaction temperature

Samples	Tafel slope ($mV \cdot dec^{-1}$)
RuO_x@ndls._40°C	135
RuO_x@ndls._80°C	111
RuO_x@ndls._100°C	98
RuO_x@ndls._120°C	83
RuO_x@ndls._160°C	71
RuO_x@ndls._200°C	100

Table S4. Electrochemical impedance spectroscopy (EIS) of catalysts with different hydrothermal reaction time.

Potential (vs. RHE)	0.05 V	0.10 V	0.15 V
RuO_x@ndls._10min	7.73 Ω	4.23 Ω	2.38 Ω
RuO_x@ndls._20min	6.08 Ω	3.61 Ω	2.22 Ω
RuO_x@ndls._40min	4.02 Ω	2.23 Ω	1.46 Ω
RuO_x@ndls._60min	2.81 Ω	1.82 Ω	1.22 Ω
RuO_x@ndls._120min	14.30 Ω	7.58 Ω	4.06 Ω

[a] The result was obtained by fitting the data using a two-time-constant equivalent circuit model.

Table S5. EIS of catalysts with different hydrothermal reaction temperature.

Potential (vs. RHE)	0.05 V	0.10 V	0.15 V
RuO_x@ndls._40°C	29.19 Ω	12.67 Ω	6.34 Ω
RuO_x@ndls._80°C	13.87 Ω	6.86 Ω	3.53 Ω
RuO_x@ndls._100°C	7.35 Ω	4.29 Ω	2.42 Ω
RuO_x@ndls._120°C	3.54 Ω	2.36 Ω	1.52 Ω
RuO_x@ndls._160°C	2.81 Ω	1.82 Ω	1.22 Ω
RuO_x@ndls._200°C	7.08 Ω	4.12 Ω	2.36 Ω

[a] The result was obtained by fitting the data using a two-time-constant equivalent circuit model.

Table S6. Tafel slopes of different catalysts

Samples	Tafel slope (mV dec^{-1})
Pt (20 wt%)/C	182
b.f.	188
ndl.	176
RuO_x@b.f.	121
RuO_x@ndls.	71

[a] The Tafel slope of RuO_x@ndls. is the smallest among the tested catalysts, indicating superior electrocatalytic kinetics.

Table S7. Tafel slopes of different metals

Samples	Tafel slope ($mV\,dec^{-1}$)
RuO_x@ndls.	71
RhO_x@ndls.	88
PtO_x@ndls.	115
IrO_x@ndls.	132
PdO_x@ndls.	129
AuO_x@ndls.	127
AgO_x@ndls.	154

[a] A smaller Tafel slope indicates faster reaction kinetics and better electrocatalytic performance.

2. Supporting Figures

List of Figures

S1. Morphology of bare foam and synthesised needles . . . 11
S2. TEM image of Cu needles, interplanar spacing is 0.30 nm. . . . 12
S3. X-ray diffraction (XRD) pattern of prepared needles on carbon paper and electrodeposited copper on carbon paper, the latter was prepared by the same method but without adding Ni. . . . 13
S4. XRD pattern of prepared needles on copper foam and XRD pattern of RuO_x@ndl. . . . 14
S5. (a) Effects of hydrothermal temperature and duration on the HER performance of RuO_x loaded on Cu foam with needle arrays. (b)Tafel slopes of catalysts with different hydrothermal reaction time. (c)Tafel slopes of catalysts with different hydrothermal reaction temperature. . . . 15
S6. EIS of catalysts with different hydrothermal reaction time, fitting by two-time-constant model equivalent circuit . . . 16
S7. EIS of catalysts with different hydrothermal reaction temperature. . . . 17
S8. Charge-transfer resistance of catalysts with different hydrothermal reaction time . . . 18
S9. Charge-transfer resistance of catalysts with different hydrothermal reaction temperature . . . 19
S10. The EDS spectra confirm that different precious metals were successfully loaded onto the catalysts, with two different sites analyzed for each catalyst. . . . 20
S11. EIS of different catalysts and their precursors. . . . 21
S12. (a) Comparison of the catalytic performance of RuO_x@f. f. with different catalyst, (b) corresponding Tafel slopes . . . 22
S13. EIS of RuO_x@b. f., RuO_x@f. f. and RuO_x@ndl. . . . 23
S14. Comparison of the Tafel slopes of RuO_x@c. p. with RuO_x/Cu_2O@c. p. . . . 24
S15. EIS of RuO_x@c. p. and RuO_x/Cu_2O@c. p.. . . . 25
S16. EIS of different noble metal catalysts. . . . 26
S17. High-angle annular dark-field imaging (HAADF) picture of RuO_x. . . . 27
S18. (a) High-angle annular dark-field imaging (HAADF) picture of RuO_x@ndl. (b) High-resolution Ru 3d X-ray Photoelectron Spectroscopy(XPS) spectra of RuO_x (The Ru 3d spectra of RuO_x were collected on a gold-coated single-crystal silicon wafer to reduce interference from the C 1s signal of the conductive carbon tape.) and RuO_x@ndl. (c) XPS survey spectrum of RuO_x (The XPS survey was conducted on conductive carbon tape to avoid interference from the gold signal.). . . . 28
S19. XRD pattern of RuO_x calcined to 500 °C during TGA analysis . . . 29
S20. Open-circuit potential of the RuO_x-loaded working electrode in the *in-situ* Raman cell . . . 30

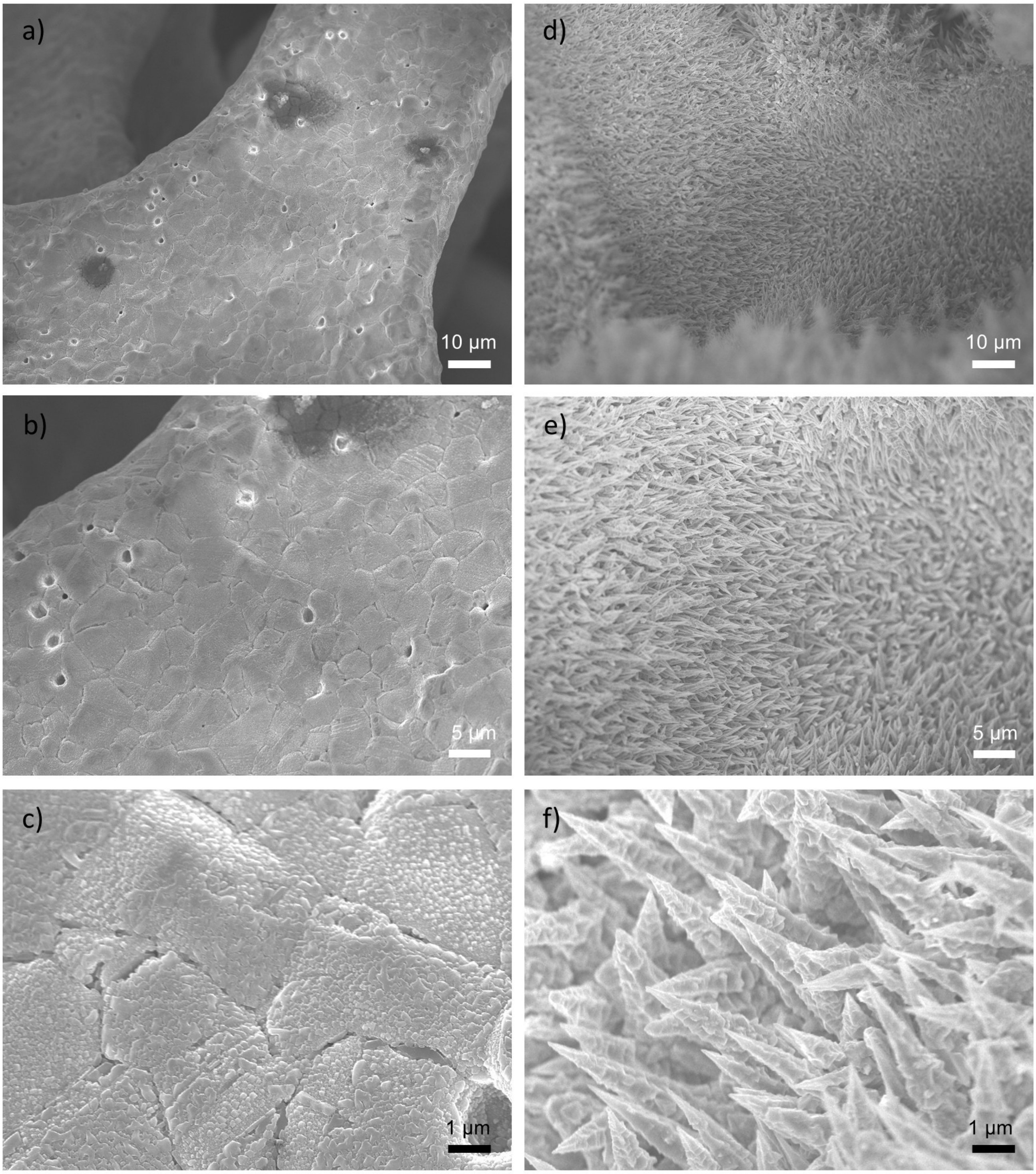

Figure S1. Morphology of bare foam and synthesised needles

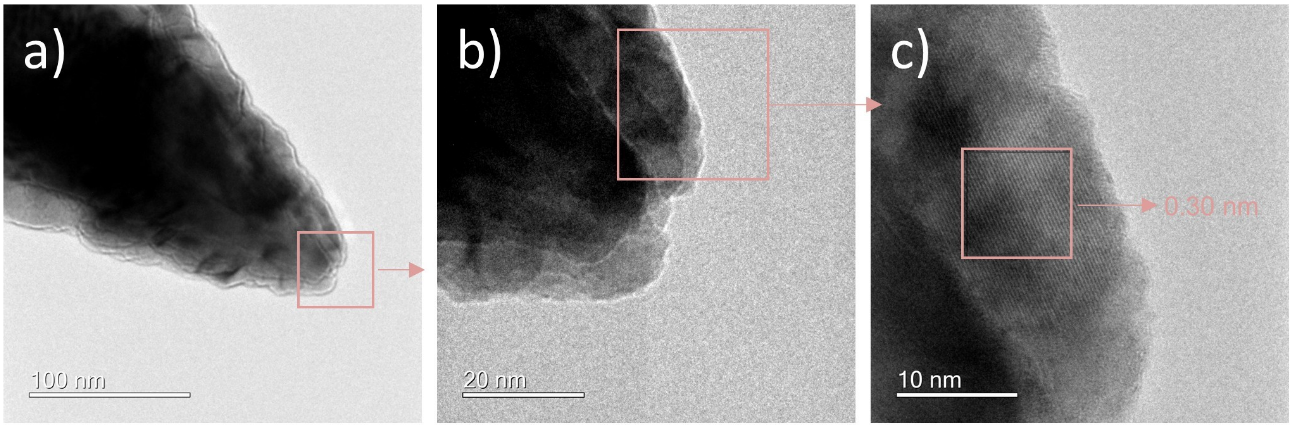

Figure S2. TEM image of Cu needles, interplanar spacing is 0.30 nm.

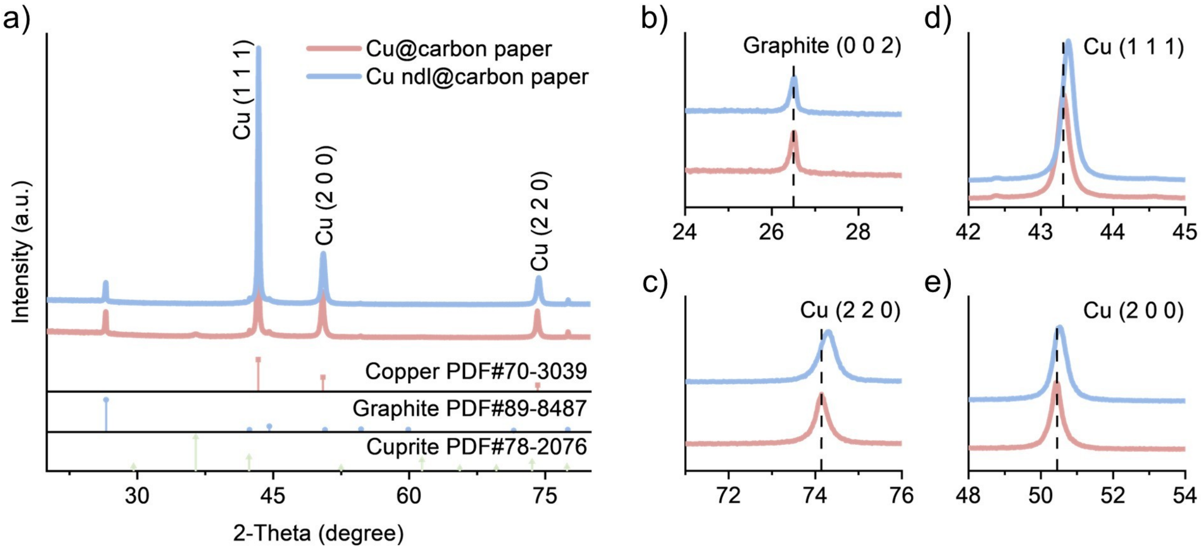

Figure S3. X-ray diffraction (XRD) pattern of prepared needles on carbon paper and electrodeposited copper on carbon paper, the latter was prepared by the same method but without adding Ni.

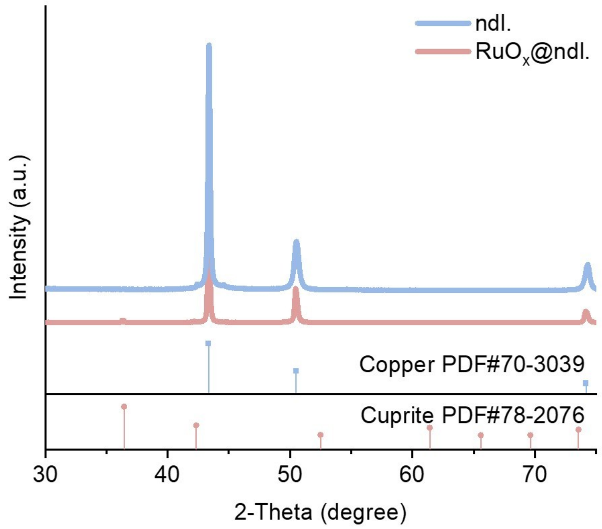

Figure S4. XRD pattern of prepared needles on copper foam and XRD pattern of RuO_x@ndl.

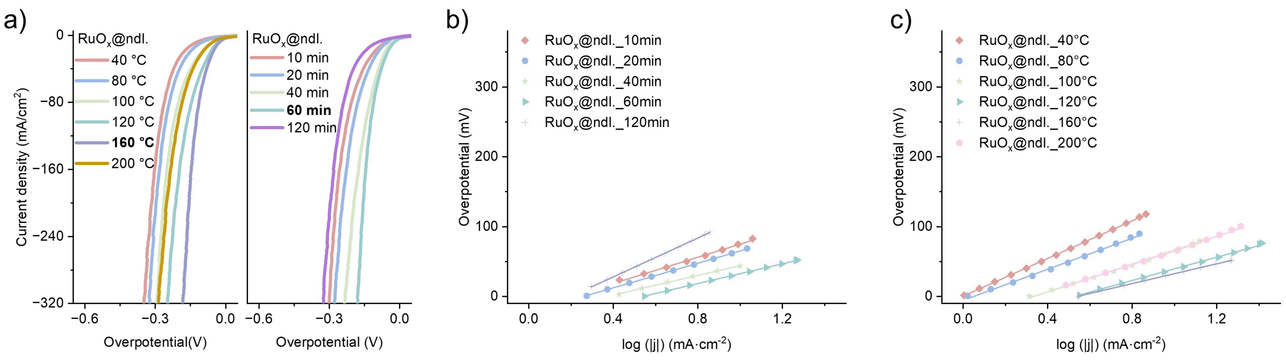

Figure S5. (a) Effects of hydrothermal temperature and duration on the HER performance of RuO_x loaded on Cu foam with needle arrays. (b)Tafel slopes of catalysts with different hydrothermal reaction time. (c)Tafel slopes of catalysts with different hydrothermal reaction temperature.

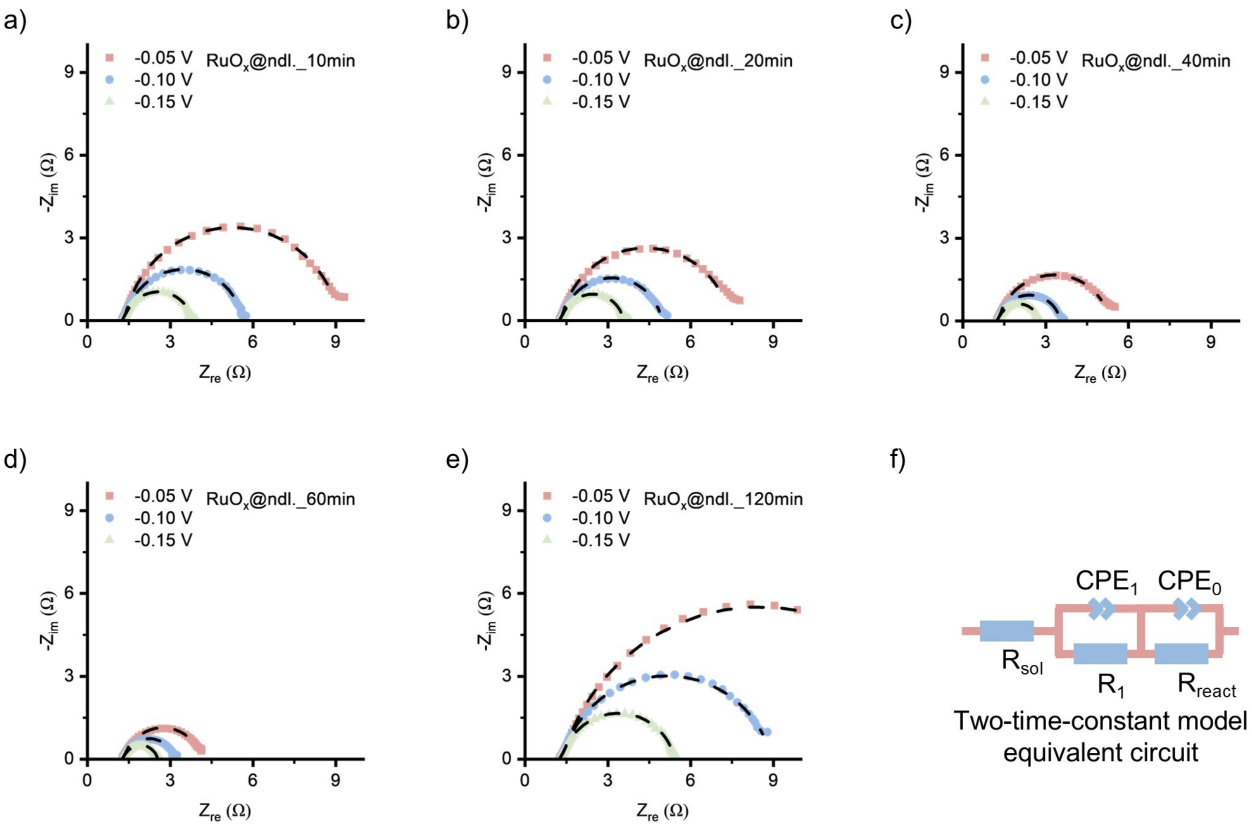

Figure S6. EIS of catalysts with different hydrothermal reaction time, fitting by two-time-constant model equivalent circuit

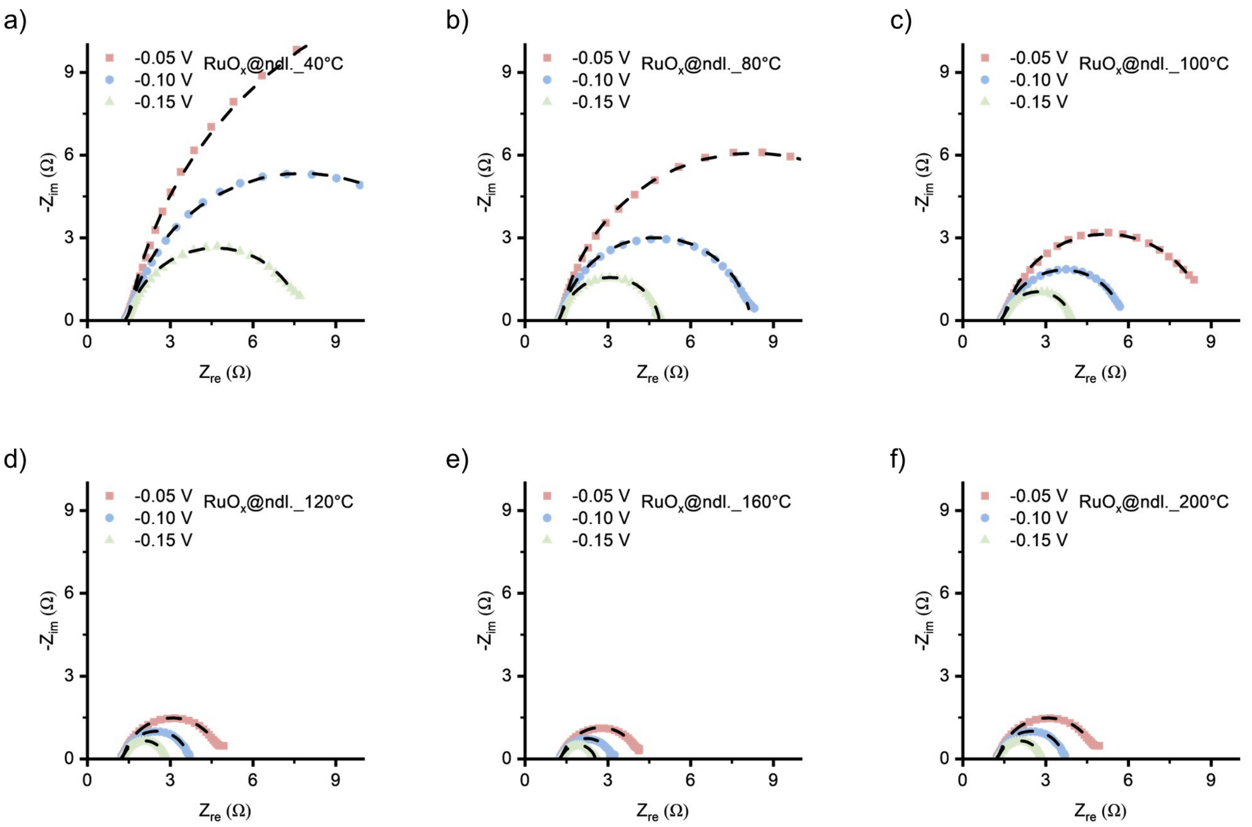

Figure S7. EIS of catalysts with different hydrothermal reaction temperature.

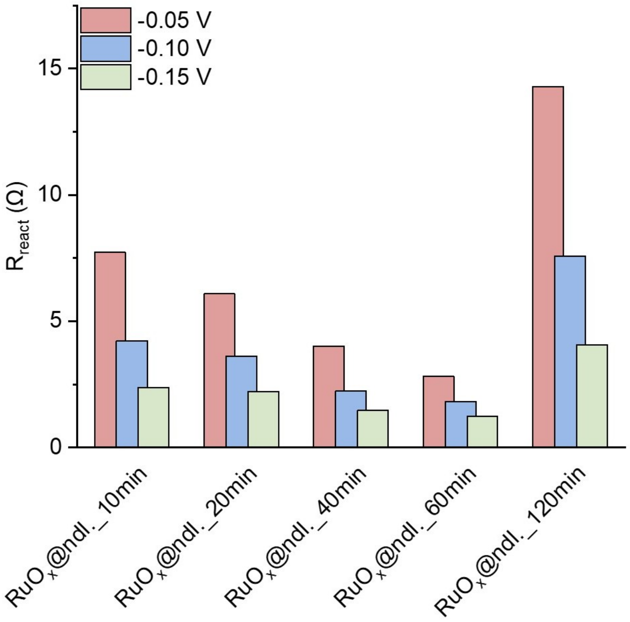

Figure S8. Charge-transfer resistance of catalysts with different hydrothermal reaction time

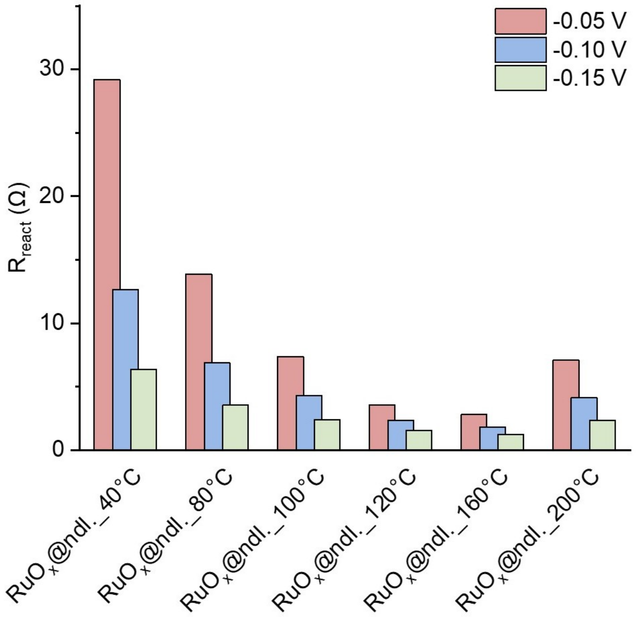

Figure S9. Charge-transfer resistance of catalysts with different hydrothermal reaction temperature

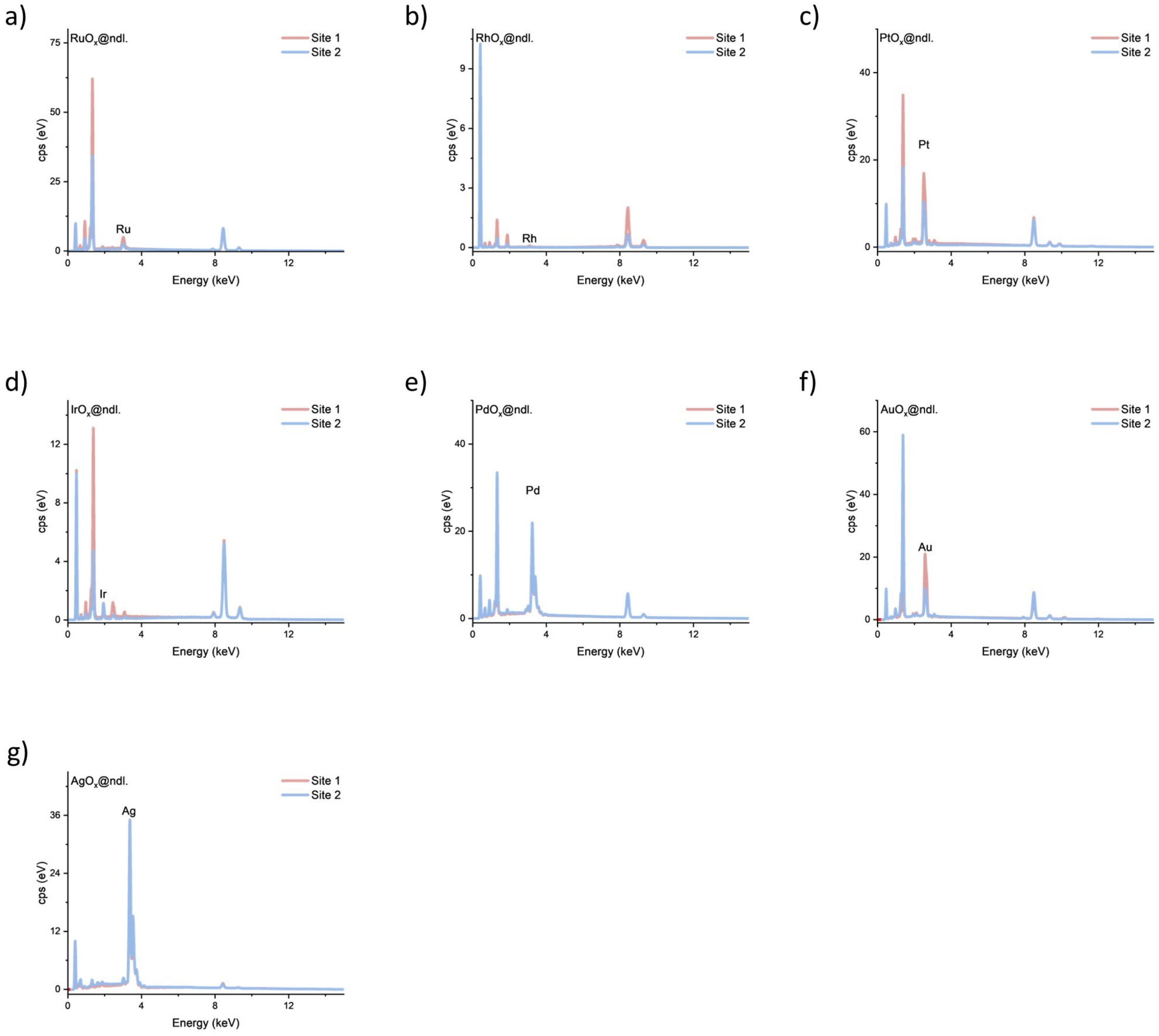

Figure S10. The EDS spectra confirm that different precious metals were successfully loaded onto the catalysts, with two different sites analyzed for each catalyst.

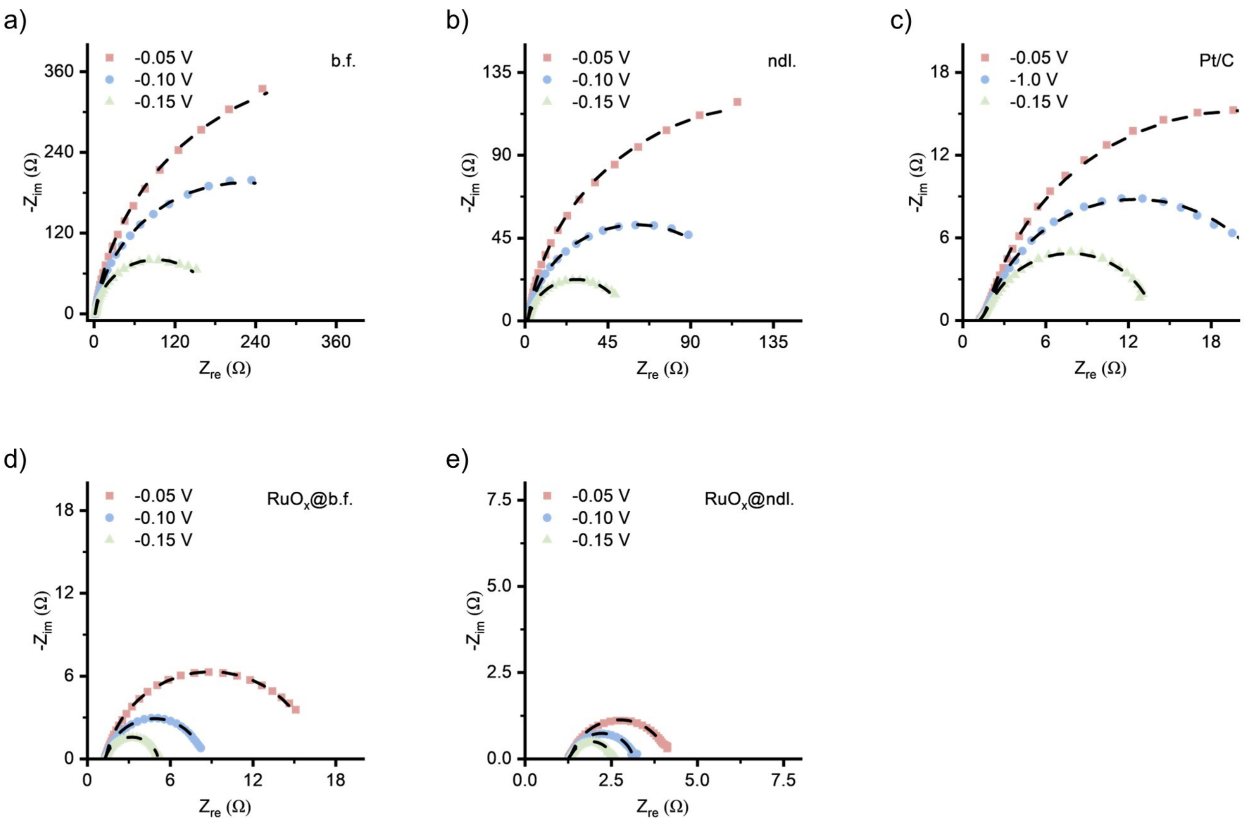

Figure S11. EIS of different catalysts and their precursors.

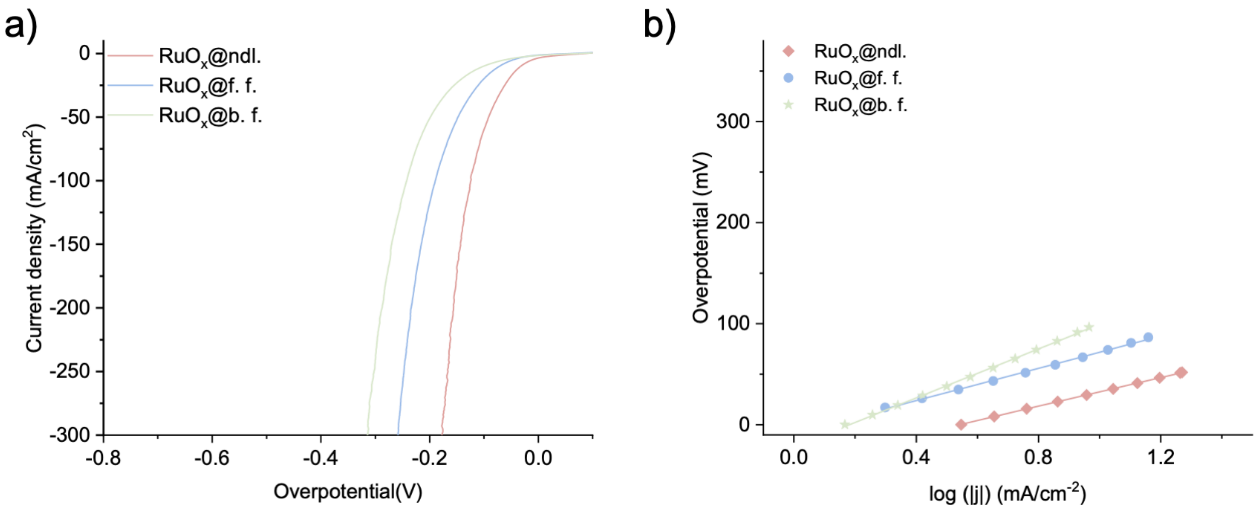

Figure S12. (a) Comparison of the catalytic performance of RuO_x@f. f. with different catalyst, (b) corresponding Tafel slopes

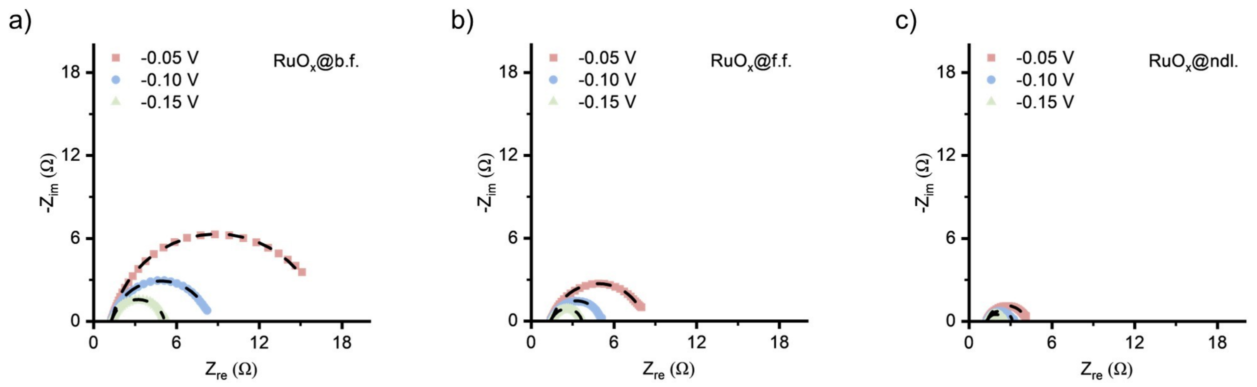

Figure S13. EIS of RuO_x@b. f., RuO_x@f. f. and RuO_x@ndl.

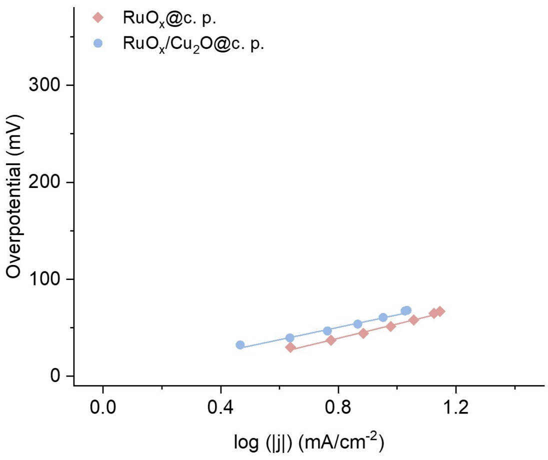

Figure S14. Comparison of the Tafel slopes of RuO_x@c. p. with RuO_x/Cu_2O@c. p.

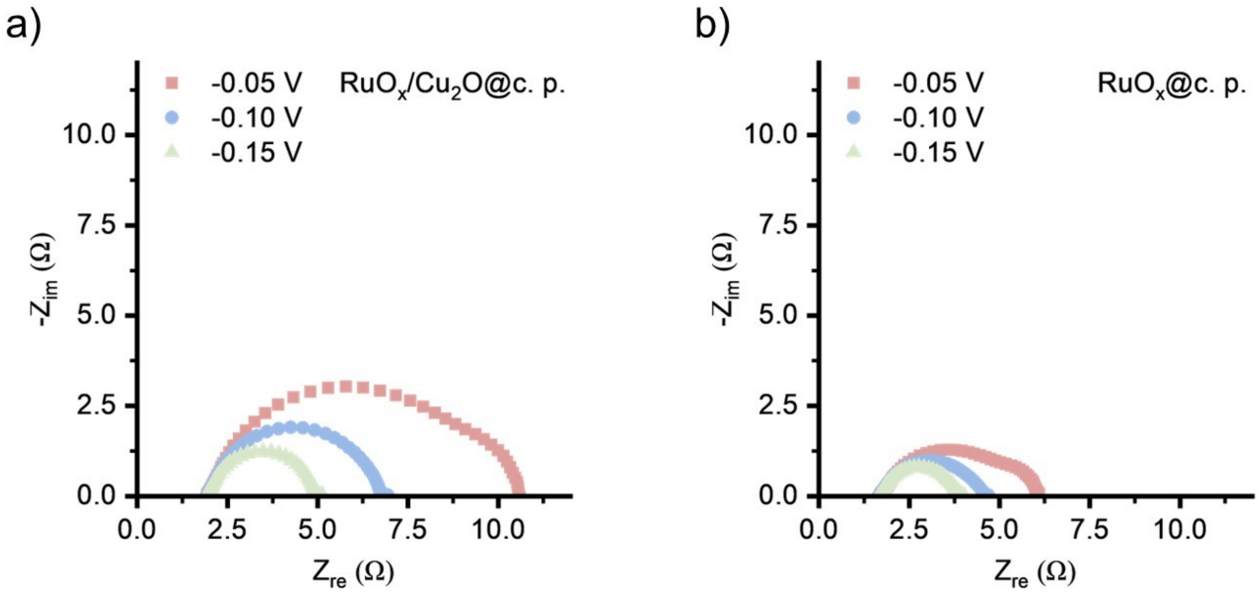

Figure S15. EIS of RuO_x@c. p. and RuO_x/Cu_2O@c. p..

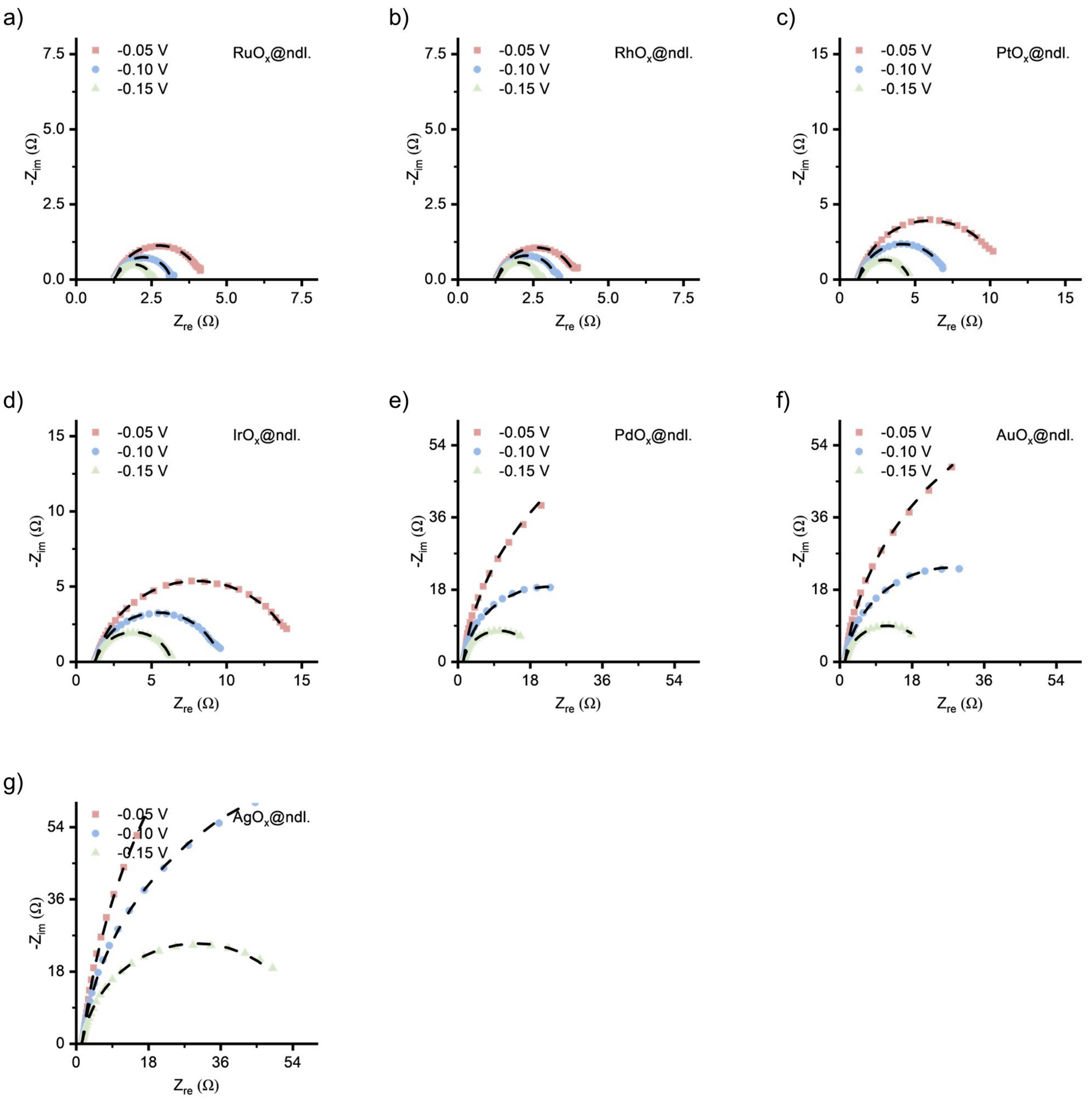

Figure S16. EIS of different noble metal catalysts.

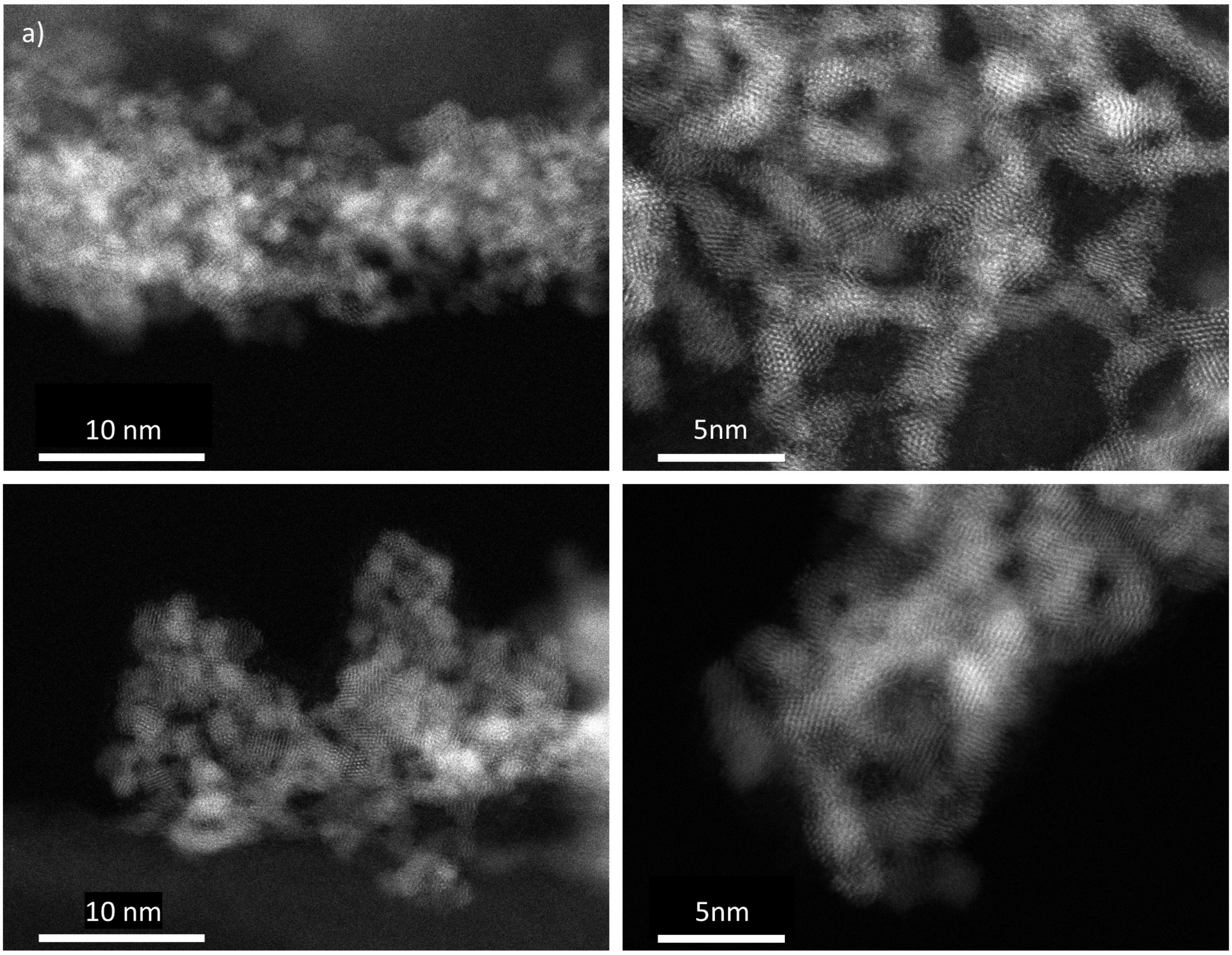

Figure S17. High-angle annular dark-field imaging (HAADF) picture of RuO_x.

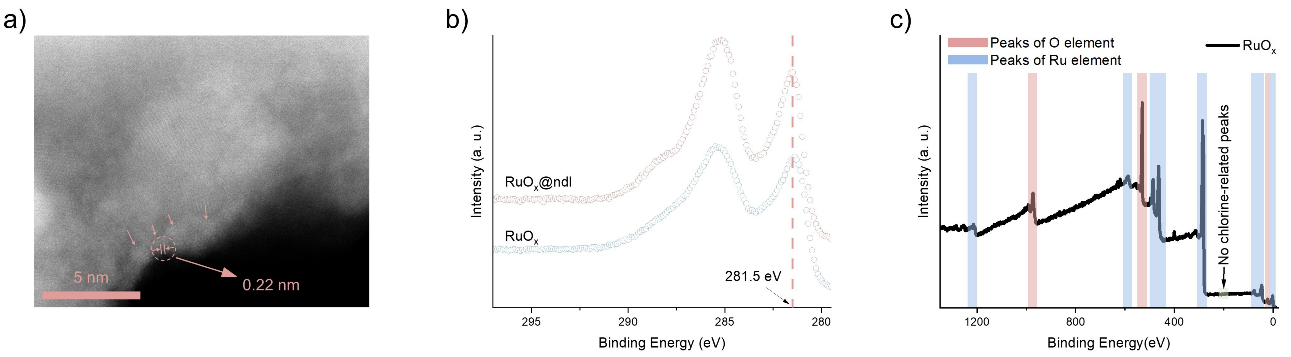

Figure S18. (a) High-angle annular dark-field imaging (HAADF) picture of RuO_x@ndl. (b) High-resolution Ru 3d X-ray Photoelectron Spectroscopy(XPS) spectra of RuO_x (The Ru 3d spectra of RuO_x were collected on a gold-coated single-crystal silicon wafer to reduce interference from the C 1s signal of the conductive carbon tape.) and RuO_x@ndl. (c) XPS survey spectrum of RuO_x (The XPS survey was conducted on conductive carbon tape to avoid interference from the gold signal.).

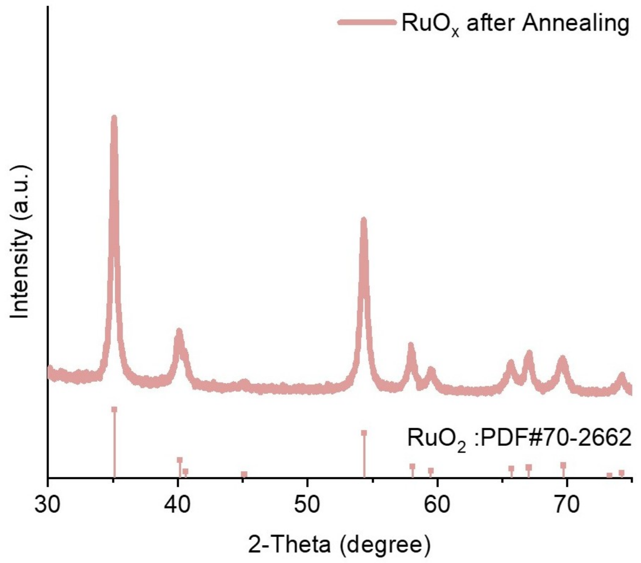

Figure S19. XRD pattern of RuO_x calcined to 500 °C during TGA analysis

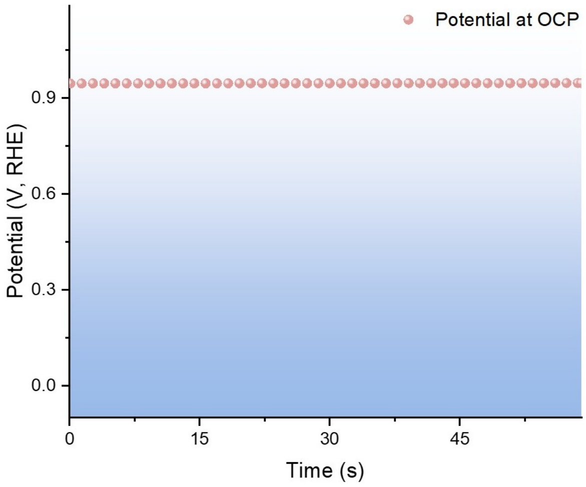

Figure S20. Open-circuit potential of the RuO_x-loaded working electrode in the *in-situ* Raman cell
